# Northwest African Neolithic initiated by migrants from Iberia and Levant

**DOI:** 10.1038/s41586-023-06166-6

**Published:** 2023-06-07

**Authors:** Luciana G. Simões, Torsten Günther, Rafael M. Martínez-Sánchez, Juan Carlos Vera-Rodríguez, Eneko Iriarte, Ricardo Rodríguez-Varela, Youssef Bokbot, Cristina Valdiosera, Mattias Jakobsson

**Affiliations:** 1 Human Evolution, Department of Organismal Biology and SciLifeLab, Uppsala University, Uppsala, Sweden; 2 Departamento de Historia, Universidad de Córdoba, Cordoba, Spain; 3 Área de Prehistoria, Departamento de Historia, Geografía y Antropología, Centro de Investigación en Patrimonio Histórico, Cultural y Natural, Facultad de Humanidades, Universidad de Huelva, Huelva, Spain; 4 Universidad de Burgos, Departamento de Historia, Geografía y Comunicaciones, Burgos, Spain; 5 Centre for Palaeogenetics, Stockholm, Sweden; 6 Department of Archaeology and Classical Studies, Stockholm University, Stockholm, Sweden; 7 Institut National des Sciences de l’Archéologie et du Patrimoine, Rabat, Morocco; 8 Department of History and Archaeology, La Trobe University, Melbourne, Victoria Australia; 9 Palaeo-Research Institute, University of Johannesburg, Auckland Park, South Africa

**Keywords:** Population genetics, Archaeology, Anthropology

## Abstract

In northwestern Africa, lifestyle transitioned from foraging to food production around 7,400 years ago but what sparked that change remains unclear. Archaeological data support conflicting views: (1) that migrant European Neolithic farmers brought the new way of life to North Africa^1–3^ or (2) that local hunter-gatherers adopted technological innovations^4,5^. The latter view is also supported by archaeogenetic data^6^. Here we fill key chronological and archaeogenetic gaps for the Maghreb, from Epipalaeolithic to Middle Neolithic, by sequencing the genomes of nine individuals (to between 45.8- and 0.2-fold genome coverage). Notably, we trace 8,000 years of population continuity and isolation from the Upper Palaeolithic, via the Epipaleolithic, to some Maghrebi Neolithic farming groups. However, remains from the earliest Neolithic contexts showed mostly European Neolithic ancestry. We suggest that farming was introduced by European migrants and was then rapidly adopted by local groups. During the Middle Neolithic a new ancestry from the Levant appears in the Maghreb, coinciding with the arrival of pastoralism in the region, and all three ancestries blend together during the Late Neolithic. Our results show ancestry shifts in the Neolithization of northwestern Africa that probably mirrored a heterogeneous economic and cultural landscape, in a more multifaceted process than observed in other regions.

## Abstract (web summary)

Genome sequencing of nine individuals shows ancestry shifts in the Neolithization of northwestern Africa that probably mirrored a heterogeneous economic and cultural landscape in a more multifaceted process than observed in other regions.

## Main

North Africa’s geographic location, centred between the vast Saharan desert, the fertile Near East and Mediterranean Europe, has resulted in a complex human history in the area^7,8^. The fossil record suggests long-term hominid and human presence^9^, although continuity over the past 100,000 years cannot be deduced due to the fragmented nature of the record. In the Late Pleistocene, 15,000 years ago, the remains of foragers excavated in Morocco show a distinct genetic make-up intermediate between contemporary Levantine foragers and sub-Saharan African populations^10^. Current-day North Africans are largely related to Eurasian populations, which was probably caused by ‘back-to-Africa’ migrations^7^.

Both archaeological records and archaeogenomic data show that Neolithic farmers (genetically distinct from European foragers) dispersed from the northern Levant and Anatolia to the Mediterranean islands, Italian peninsula and Iberia^11–18^. Mediterranean coastal routes have long been recognized in the archaeological record as an important part of the Neolithic expansion in Europe. In the western Mediterranean, Impressed Ware technology—and further the Cardial Horizon—spread along the European mainland coast and islands to reach the Iberian peninsula, where both phenomena are present at 7,550 calibrated years before the present (cal bp) (refs. ^19,20^).

Whereas some studies support a simultaneous appearance of the Neolithic in northwestern Africa (Eastern Rif, Ifri Oudadane site) and Iberia around 7,550 cal bp (ref. ^21^), the earliest evidence for pottery, domestic cereals and husbandry is found in northern Morocco approximately two centuries later at Kaf Taht el-Ghar (KTG) around 7,350 cal bp (refs. ^2,3,22,23^). Although Early Neolithic material culture and the first domestic mammals and pulses suggest a connection to Iberia^1–3^, the extent and legacy of these connections remain unclear. However, the first genomic analysis of Early Neolithic farmers from northwestern Africa (from the site Ifri n’Amr o’Moussa (IAM) in central Morocco) shows no traces of admixture with European Neolithic farmers. Instead, it shows long-term population continuity since the Upper Palaeolithic in the region^6^. This result aligns with the hypothesis that the Neolithic transition in northwestern Africa was initiated by local Epipalaeolithic communities adopting technological innovations^4,5^, such as those found at IAM: impressed Cardial-like ceramics, similar to those present throughout the western Mediterranean Neolithic Europe, and domestic cereals (for example, a grain of *Hordeum vulgare* dated around 7,050 cal bp)^2^. This pattern implies a Neolithization process that contrasts markedly with that of Europe, where it has been established that agriculture was introduced by the west- and northward demic diffusion of Anatolian early farmers^11,12^. The local development, or acculturation, of the North African Neolithic is further supported by signs of increasingly sedentary Epipalaeolithic groups developing strategies for resource management, such as the exploitation of wild plants and pottery^1,4,24–26^. Rapid climatic changes favoured mobile herding^27^ and, whereas it has been hypothesized that cattle were independently domesticated in the Sahara^28^, radiocarbon data suggest a gradual introduction of pastoralism in the Sahara in a southwestwards direction 7,000–6,000 cal bp, possibly from the Near East^29,30^.

Whereas palaeogenomic studies on the European Mediterranean Neolithic transition are abundant^15,31–33^, North Africa has been the focus of only a single study that generated human genetic data from one Early and one Late Neolithic site^6^, leaving substantial gaps in the chronology of events. It is evident that the site of IAM shows a Neolithic lifestyle and an absence of European Neolithic ancestry, but whether this was an independent development or the inspiration came from other groups in northwestern Africa or across the Mediterranean Sea remains unclear. Hence, the timeline and processes involved in the Neolithization of the region, the nature and dynamics of different economies in North Africa and the role they may have played in the broader European Neolithic remain understudied and controversial.

In this study we investigate a time series of human remains from four archaeological sites spanning the Epipalaeolithic to Middle Neolithic in current-day Morocco: the Epipalaeolithic site of Ifri Ouberrid (OUB), the Early Neolithic sites of IAM and KTG and the Middle Neolithic cemetery of Skhirat-Rouazi (SKH), co-analysed with previously published genetic data from that region^6,10^. By sequencing the genomes of nine individuals excavated from these four archaeological sites, we can demonstrate that the Neolithic transition in northwestern Africa was ignited by migration of Neolithic farmers from Mediterranean Europe.

**Fig. 1:**
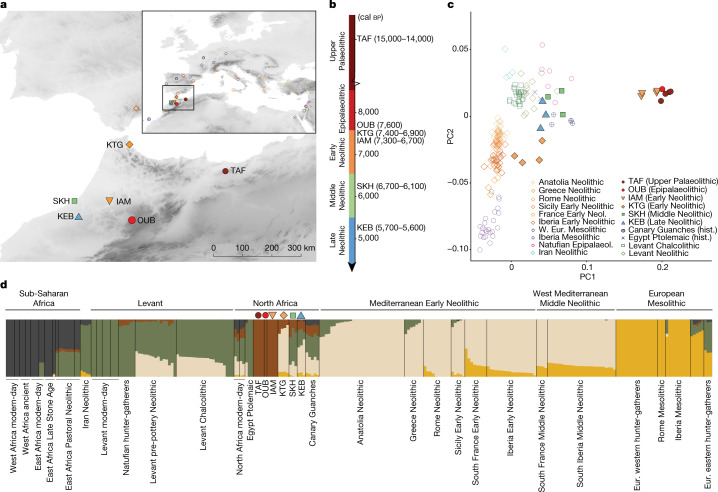
Overview of ancient northwestern African genetic composition. **a**, Geographic location of investigated archaeological sites. Symbol legend given in **c**. The map was generated using the open source QGIS Geographic Information System, http://qgis.osgeo.org. **b**, Chronological representation of the investigated archaeological time periods of northwestern Africa, with each site’s radiocarbon-dated timeline indicated. **c**, Enlarged view of a PCA plot (Supplementary Fig. 3) with focus on the ancient individuals analysed. Each projected ancient individual is represented by a coloured symbol. W. Eur., West European; hist., historical. **d**, Estimated ancestry proportions for relevant African, Middle Eastern and European (Eur.) modern-day and ancient individuals (assuming five ancestry components; additional results are presented in Supplementary Fig. 4). Pre-Neolithic and Neolithic northwestern African populations/individuals are highlighted by the same symbols used in **a** and **c**.

We generated genomic sequence data from nine ancient individuals from modern-day Morocco (Table 1), ranging in genome coverage from 45.75- to 0.017-fold, including five individuals with more than onefold coverage and three with more than ninefold. Chronologically the data span more than 1,000 years, covering the Late Epipaleolithic (*n* = 1), Early Neolithic (*n* = 5) and Middle Neolithic (*n* = 3). Two Early Neolithic sites were studied—KTG (*n* = 4) and IAM—where we co-analysed the newly generated genomic data of one individual and those previously reported^6^ (Fig. 1a,b). DNA libraries were generated from DNA extracts obtained from bones and teeth and subsequently shotgun sequenced on an Illumina platform. All libraries presented the degradation patterns expected from ancient DNA, including short fragment sizes and cytosine deamination at read ends (Supplementary Fig. 1). Contamination estimates were generally low for both the nuclear genome and mitochondria except for individual skh003, which showed 10–16% nuclear contamination (Table 1). To assess the relationship of the ancient northwestern African individuals to other ancient and present-day West Eurasian and African populations, we co-analysed our data with relevant ancient (Supplementary Data 2) and current-day groups from Africa, the Middle East and Europe^34^.

**Table 1:** Summary information of archaeological and newly generated genomic data from the ancient individuals reported in this study

Individual	Archaeological site	Archaeological association	cal bp 94.5%	Genome coverage	mt Coverage	Sex	mt Haplogroup	Y haplogroup	Autosomal contamination (%)
oub002	OUB	Epipalaeolithic	7660–7506	45.760	2853.42000	XX	U6a6b	–	1.0440
ktg001	KTG	Early Neolithic Cardial	7423–7267	0.0170	1110.31000	XY	U6	^a^	0
ktg004	KTG	Early Neolithic Cardial	7159–6945	9.020	2819.18000	XY	HV0 + 195	G2a2b2a1a1c1a	2.0035
ktg005	KTG	Early Neolithic Cardial	7429–7285	1.740	988.41400	XX	U5b2b1a	–	1.7870
ktg006	KTG	Early Neolithic Cardial	7247–6995	1.300	253.99100	XY	J1c3j	G2a2b2a1a1c1a	0.5980
iam004 (IAM.1^b^)	IAM	Early Neolithic	6894–6679^b^	0.270	8.92969	XX	U6a7	–	0
skh001	SKH	Middle Neolithic	6437–6295	9.180	492.87900	XX	M1a1b	–	2.5360
skh002	SKH	Middle Neolithic	6733–6500	0.960	64.96840	XY	J2a2d	T1a1a	2.0610
skh003	SKH	Middle Neolithic	6298–6121	0.086	20.69170	XY	U6c	T1a1a	10.8400

The summary includes archaeological site names, chronological archaeological association, radiocarbon dating estimates (cal bp), average genome coverage, average mitochondrial (mt) genome coverage, mt and Y chromosome haplogroups and contamination estimates based on autosomes. Calibrated dates from atmospheric curve IntCal20 (ref. ^41^).

^a^Insufficient coverage. ^b^Individual previously reported and radiocarbon dated in ref. ^6^.

## Eight thousand years of population continuity

From the Upper Palaeolithic people of Taforalt (TAF) via the Epipalaeolithic at OUB to the Early Neolithic at IAM, we observe the persistence of the unique genetic make-up that existed in northwestern African inhabitants 15,000 years ago (Fig. 1c,d and Supplementary Fig. 5), and possibly even further back in time. The Epipalaeolithic individual oub002, dating to 7,660–7,506 cal bp, is genetically very similar to individuals from TAF (15,086–14,046 cal bp)^35^ and Early Neolithic individuals from IAM (7,316–6,679 cal bp; Fig. 1)^6,36^. The genome of Oub002 demonstrates a marked population continuity in northwest Africa with no substantial gene flow across the Mediterranean Sea for at least 7,000 years across the Epipalaeolithic (Fig. 1c,d), linking the Maghrebi genetic ancestry found in the Upper Palaeolithic to the Early Neolithic individuals at IAM.

**Fig. 2:**
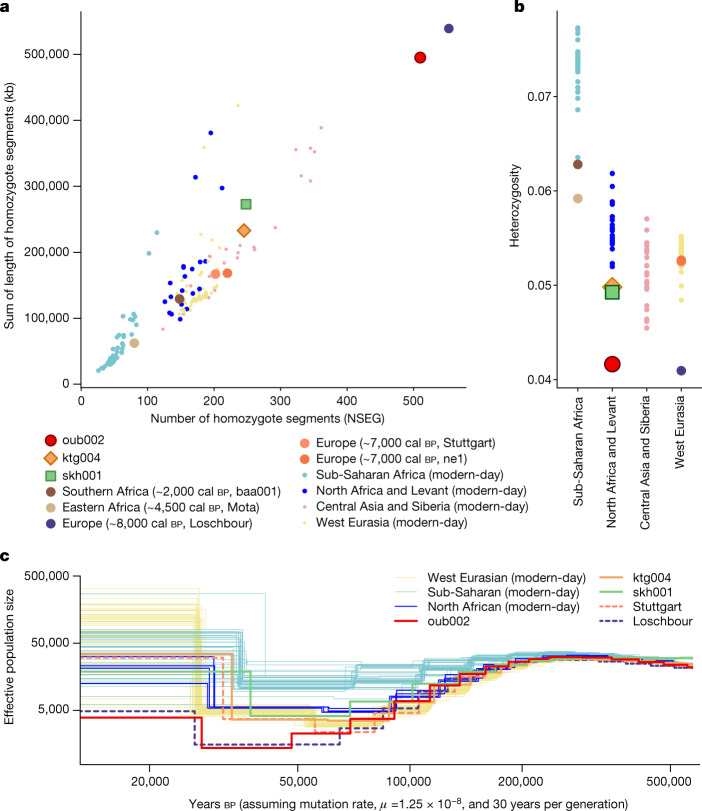
Measures of genetic diversity in ancient (northwestern and sub-Saharan) Africans and Eurasians, computed using diploid calls from higher-coverage (over ninefold genome coverage) individuals. **a**–**c**, Ancient individuals (including oub002, ktg004 and skh001) are compared with modern-day individuals from geographically corresponding regions. **a**, Runs of homozygosity. ne1, Neolithic European 1; NSEG, number of homozygote segments. **b**, Heterozygosity, calculated from the number of variable positions per individual divided by the number of single-nucleotide polymorphism (SNP) sites per individual. **c**, Effective population size over time, as inferred by pairwise sequentially Markovian coalescent, for three ancient northwestern Africans with over ninefold genome coverage, as well as for a Mesolithic European individual (Loschbour) and a Neolithic European individual (Stuttgart), and modern-day individuals for comparison.

The Maghrebi lineage shows outstandingly low genetic diversity^6,10^ (Fig. 2b and Supplementary Fig. 9) and long and frequent runs of homozygosity (RoH) (Fig. 2a), probably as a consequence of long-lasting isolation. By investigation of the 45.8-fold genome of oub002 we show that ancient northwestern Africans went through a severe population bottleneck. Until some 70,000–60,000 years ago the effective population size (*N*_e_) changes of oub002 follow a pattern similar to that of Eurasian populations with a relatively small effective population size reached 50,000 years ago (Fig. 2c), which is consistent with the Maghrebi lineage being related to the populations that migrated out of Africa. Interestingly, modern-day Eurasians and North Africans, as well as Neolithic Eurasians effective population size remained at around 5,000 until about 30,000 years ago but the effective population size of the Maghrebi lineage continues to decrease and reached its lowest point (*N*_e_ ≈ 1,400) between 50,000 and 27,000 years ago during the peak of the Last Glaciation. Remarkably similar patterns are observed for the Mesolithic western hunter-gatherers (WHG) of Europe (represented by Loschbour in Fig. 2c), for which low diversity measures have been attributed to high levels of background relatedness and autozygosity due to small population size^37^.

## European farmers induce Neolithization

At the site of IAM, a multitude of artefacts representing the Neolithic package have been identified. However, it has been shown that the people living at IAM show autochthonous Maghrebi ancestry^6^ and were the descendants of earlier (Upper Palaeolithic and Epipaleolithic) northwestern African groups (Fig. 1c,d). These two observations support the view that the first stage of the Neolithic transition in Morocco was driven by local populations adopting technological innovations based on contacts across the Mediterranean^2^.

**Fig. 3:**
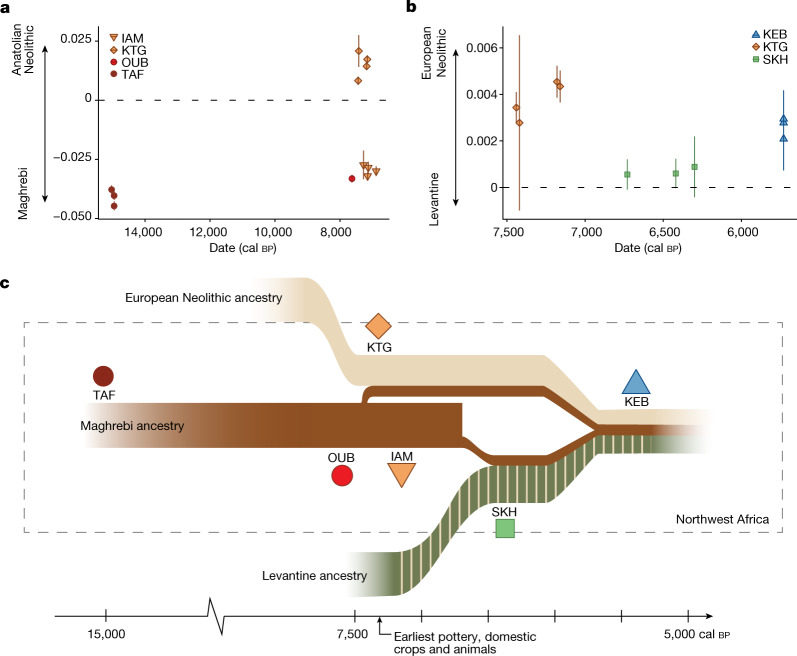
Genetic affinities of Stone Age northwestern Africans and schematic summary of the population history of the Maghreb. **a**, Genetic affinity of analysed Stone Age northwestern African individuals, polarized between Neolithic Anatolia and Maghrebi ancestry, using the *f*_4_ test of the form *f*_4_(Anatolia Neolithic, TAF011; Stone Age northwestern African individuals, Mbuti). **b**, Genetic affinity of Stone Age northwestern African individuals, polarized between Early Neolithic Iberian and Levantine ancestry using *f*_4_ test of the form *f*_4_(Iberia Early Neolithic, Levant Chalcolithic; Neolithic northwestern African individuals, Mbuti). **a**,**b**, Each symbol represents a single individual *f*_4_ value. Error bars indicate ±2 s.e., computed with a block jack-knife approach (5 Mb blocks weighted by the number of SNPs). **c**, Summary of inferred population history of the Stone Age Maghreb.

The Early Neolithic site of KTG, located on the North African Mediterranean coast near the Gibraltar strait (Fig. 1a), predates and partly overlaps in time with IAM^2^ (Table 1). At KTG a full Neolithic assemblage is found, including a diversity of cultivated cereals, domestic mammals and cardial ceramics^38,39^. In contrast to the people at IAM, those at KTG are genetically similar to European Early Neolithic populations (Figs. 1c,d and 3a). Interestingly, all four KTG individuals show admixture (15.4–27.4%) with local North African groups (Fig. 1d), consistent with significantly positive values for the *f*_4_ test of admixture (KTG, Mediterranean EN; TAF, Mbuti) (Supplementary Data 7). Furthermore we identify a small proportion of WHG ancestry in KTG (Fig. 1d), consistent with the observation of Early Neolithic Europeans carrying WHG ancestry^14,15,31,33,40^. A population history model for the KTG people with 72 ± 4.4% Anatolian Neolithic ancestry, 10 ± 2.6% WHG ancestry and 18 ± 3.3% Maghrebi ancestry is consistent with the data (qpAdm, *P* = 0.193). Taken together, these results suggest a European Neolithic origin of KTG farmers whose ancestors dispersed from Anatolia throughout Europe, admixing with European hunter-gatherers on their path to southwestern Europe^33,40^ before crossing the Mediterranean to North Africa. The presence of European hunter-gatherer ancestry excludes the possibility that Early Neolithic migrants exclusively followed North African Mediterranean shores from Anatolia or the Levant.

Iberian Early Neolithic (both as a whole and regionally) was found to be the best source population for the European ancestry in KTG, followed by Sicily Stentinello Early Neolithic (Supplementary Data 9). This is consistent with low levels of genetic differentiation in Cardial Ware-associated groups along the European shores of the Mediterranean Sea^41^, confirmed by direct radiocarbon dates showing that Impressed Ware farmers expanded rapidly across the western Mediterranean^3,19,42^.

It has been debated whether European farmers crossed from Iberia to Morocco^2,3^ or whether earlier crossings of the Mediterranean would have happened, through the Sicilian–Tunisian Strait followed by a Maghrebi route of expansion^4,43^. Direct comparisons of Early Neolithic farmers from Sicily and Iberia as ancestors of KTG farmers provide stronger evidence for an Iberian Neolithic origin (Supplementary Data 9 and Supplementary Information 8), but we cannot exclude some contribution from Sicilian farmers. Genetic data are consistent with the most parsimonious explanation for archaeological evidence on the Neolithic transition in northwestern Africa: the crossing from southern Iberia by Iberian Neolithic farmers^2,23^. The close geographical proximity between southern Iberia and the Tangitana Peninsula adds strength to this observation whereas the lack of reliable archaeological evidence of early domestic elements in relevant sites along the eastern Maghreb and Tunis, including sites with pottery and obsidian from Pantelleria Island, undermines the Sicily–Tunis crossing hypothesis^3^. Interestingly, gene flow from North Africa was found only in Mediterranean European individuals much later, from around 4,500 years ago^31,44^.

Different individuals from KTG date to slightly different time periods. We find a twofold larger proportion of Maghrebi ancestry in earlier KTG individuals (roughly 25%, ktg001 and ktg005, approximately 7,429–7,267 cal bp) than in later ones (about 13%, ktg004 and ktg006, around 7,247–6,945 cal bp) (Fig. 1d). This coincides with an increase in European Neolithic ancestry, shown by the significantly negative result for *f*_4_(KTG earlier, KTG later, Iberia Early Neolithic, Mbuti; *z*-score = −5.01). Approximately one quarter of Maghrebi ancestry in early KTG suggests that they represent at least the second generation of interbreeding between the groups. We estimated the time of admixture using two approaches based on ancestry covariance patterns and linkage disequilibrium decay, using Iberia or Sicily Early Neolithic and TAF as admixture sources. Both methods date the contact within the last six to 13 generations (Supplementary Information 8), suggesting that mixing between groups occurred for a few hundred years, which is consistent with analysis of pottery style that points to the first contact at 7,500–7,400 cal bp (ref. ^23^).

Kaf Taht el-Ghar farmers had slightly lower genetic diversity levels and greater RoH than most Early Neolithic European populations (Fig. 2a,b and Supplementary Fig. 9). The Maghrebi ancestry carried by KTG people shows markedly lower diversity and more extensive RoH, and is probably the cause of the reduction in overall diversity. Archaeological evidence suggests that Early Neolithic farming was restricted to enclaves in westernmost Maghreb, possibly due to climatic constraints to the south^4,22^. This could have limited the potential of these groups to recover from an initial founder effect.

Overall, the genetic patterns of local interaction between different groups in northwestern Africa are comparable to those found in Europe: farmers assimilated local foragers’ ancestry in a unidirectional admixture process. Cases of hunter-gatherer communities adopting certain elements of the Neolithic have been described in Europe^11,14,45^. However, the northwestern Africa Neolithization process involved the notable survival of genetically unadmixed local populations (represented by IAM), despite coexisting for at least 300 years with foreign farming communities (KTG), and still adopted several elements of the Neolithic ways of living from them. Whereas the archaeological findings in IAM and KTG point to the exchange of ideas between groups and support an acculturation process of foraging communities^1,4^, our genetic data show that the exchange of genes was unidirectional.

## Influx of Levantine ancestry

Another, distinct, ancestry was introduced to northwestern Africa during the Middle Neolithic. All individuals from SKH show large proportions of a genetic component maximized in individuals from Neolithic and Chalcolithic Levant, Ptolemaic Egypt and modern-day Near Eastern populations (Fig. 1d). The ancestry in SKH can be modelled as a two-way admixture between Levant Neolithic populations (roughly 76.4 ± 4.0%) and local northwestern Africans (represented by TAF; 23.6 ± 4.0%). If a European Neolithic (for example, from Iberia) additional source population is added, the model is rejected.

Because this Neolithic Levantine ancestry has not been observed on the European side of the Mediterranean during the Neolithic, it probably represents an independent expansion of people from the Levant into North Africa. Migrations from the Levant to eastern Africa have been identified for Neolithic pastoralist individuals around 4,000 years ago, who are presumed descendants of unsampled northeastern African populations associated with the spread of Saharan pastoralism^46^. Both in SKH and eastern African Neolithic pastoralists, Levantine ancestry is admixed with local ancestries (Fig. 1d, Supplementary Information 8 and Supplementary Data 12). The arrival of this Levantine ancestry coincides with the appearance of a new ceramic tradition in northern Morocco, often characterized by cord-impressed motifs (‘roulette’ or wavy line), like the grave goods at Skhirat belonging to Ashakar Ware pottery^47,48^. In parallel, cattle pastoralism was expanding in the current Sahara territory^30,47^ and Afro-Asiatic language groups spread throughout the whole of North Africa^22^.

Our analyses show that the Levantine-associated component also remains in the Maghreb during the Late Neolithic in individuals from Kehf el Baroud (KEB) and in the Guanches of the Canary Islands (around 1,000 cal bp; Fig. 1c,d)^6,49^. Individuals from these sites are shifted towards ancient Levantine populations on the principal component analysis (PCA) space (Fig. 1c). This highlights the complex demographic processes that took place in northwestern Africa, in contrast to the gradual increase in hunter-gatherer ancestry described in Middle and Late Neolithic Europe^32,33,40^.

The Late Neolithic individuals from KEB can be modelled as a mix of ancestries already present in northwestern Africa during the Early Neolithic and Middle Neolithic, suggesting that there were no waves of substantial migration into this region between the Middle Neolithic and Late Neolithic (Supplementary Information 8 and Supplementary Data 13).

## Conclusion

The complex population structure in modern-day northwestern Africa has been linked to various historical events, such as the Arab expansion^7,8^. However, our detailed chronology and high-resolution genomic data provide a new understanding of these prehistoric processes in the Maghreb and unveil a rich and diversified genetic substrate with Neolithic origin. First, human populations in northwestern Africa show genetic continuity and isolation since the Upper Palaeolithic, from at least 15,000 to around 7,500 years ago, when this period of isolation was interrupted by the migration of European Early Neolithic groups introducing farming practices. Hence, despite a relatively small geographic distance between southern Iberia and northwestern Africa (the distance today is only 13 km across the Gibraltar straight), and the fact that both regions were populated by foragers for many millennia prior to the Neolithic, gene flow across the Mediterranean Sea was not established until the Early Neolithic. The newcomers brought new ways of life, farming practices, domestication and pottery traditions that were subsequently adopted by local populations. Our results show that the Neolithization process in northwestern Africa was ignited by migrant Neolithic Europeans, but that local groups (at least the individuals analysed at IAM) adopted some of these practices without mixing with the newcomers. Two genetically distinct groups coexisted in close proximity in the region. Interestingly, cultural and technological knowledge appear to have been transferred mainly from European Neolithic farmers to local groups (for example, at IAM) whereas genetic ancestry flowed only from local groups to the incoming farmers, such as the population of KTG. Furthermore, in the Middle Neolithic a new ancestry with an eastern origin is detected in northwestern Africa. This ancestry indicates new migrating groups, potentially associated with Sahara pastoralists, which admixed with local groups (Fig. 3c).

The various waves of migration and admixture into northwestern Africa during the Neolithic possibly resulted in a heterogeneous economic and cultural landscape in that region—a mosaic of groups that included incoming farmers from Iberia, foragers adopting farming practices and eastern pastoralists admixing with local people. Most of these groups showed reduced effective population size and lower diversity than the contemporary populations in Europe (Fig. 2), suggesting that population sizes remained modest throughout the Neolithic. These patterns were probably caused by periods of isolation, which may have contributed to the distinct genetic ancestry seen in the Maghreb today. A recent study from the Iron Age suggests that northwestern Africa remained home to a diverse set of groups throughout prehistory^50^, making this part of the world one of the most unique places to have been studied with the archaeogenomic toolkit.

## Methods

Detailed descriptions for each section can be found in Supplementary Information.

### Archaeological sampling

The ancient human remains analysed in this study derive from a scientific cooperation agreement between INSAP, La Trobe and Uppsala Universities. Complete bone and teeth elements were brought to the ancient DNA facility in Uppsala, Sweden for further cleaning and sampling.

### Radiocarbon dating

All individuals investigated were directly radiocarbon dated at the Tandem Laboratory, Uppsala, except for ktg001, who was dated at the Beta Analytic Carbon dating laboratory, and iam004, who’s date was obtained from ref. ^6^. Radiocarbon calibration for newly reported and relevant previously published dates was performed using Oxcal v.4.4 and the IntCal20 dataset^41^.

### Ancient DNA retrieval

Human remains were sampled in dedicated clean-room laboratories at Uppsala University, Sweden after a series of stringent procedures aimed at minimization of bone and tooth surface contamination. Thirty to sixty milligrams of bone powder or solid pieces of bone material were used for DNA extraction either following ref. ^51^, with adaptations as described in ref. ^15^, or following ref. ^52^, with adaptations to the binding buffer, and an initial predigestion step with 1 ml of 0.5 M EDTA pH 8.0 for 30 min at 37 °C^53^. Sample digestion was performed overnight with 1 ml of 0.45 M EDTA pH 8.0 and 0.2 mg ml^–1^ proteinase K. Double-stranded, blunt-end-repaired DNA libraries were built with ligated P5 and P7 adaptors^54^. After assessment of DNA authenticity, quality and quantity (by estimation of endogenous DNA content, post mortem deamination patterns and fragment size distribution), the remaining DNA extract (for samples with over 1% proportion of human DNA) was used to build four to six additional double-stranded DNA libraries; for extracts with roughly 5% endogenous human content or more, 15–20 µl of DNA extract was treated with uracil DNA glycosylase (UDG) for double-stranded library building^55^. Libraries were PCR amplified using a unique 7 bp indexed primer^54,56^ in either four reactions of 25 µl or two of 50 µl, with the application of 12–20 PCR cycles depending on previous qPCR quantification cycle indication. Two extraction negative controls, two library negative controls and one PCR negative control were included per sample batch. PCR reactions were pooled and purified with AMPure XP beads (Agencourt, Beckman Coulter). Library quality was checked by electrophoresis on Tapestation (Agilent High Sensitivity D1000 ScreenTape, Agilent) and DNA concentration was quantified using a Qubit dsDNA HS (High Sensitivity) Assay Kit (Invitrogen). Equimolar pools of amplified and purified libraries were sequenced on Illumina HiSeq X at the SNP & SEQ Technology Platform in Uppsala. To reach higher coverage, between four and ten libraries were pooled equimolarly and sequenced to depletion.

### Bioinformatics data processing and authentication

Data were demultiplexed according to the indexed primer sequence and adaptors were trimmed with either MergeReadsFastQ_cc.py^57^ or Adapter Removal v.2.1.7 (ref. ^58^). Forward and reverse paired-end reads were merged when an overlap of at least 11 bp was found. Mapping against the human reference genome build 37 (hs37d5) was done using Burrows–Wheeler aligner 0.7.13 (ref. ^59^). For each library we merged bam files resulting from all resequencing rounds using SAMtools merge v.1.5 (ref. ^60^). We then separately merged data from UDG-treated and untreated libraries for each individual and used data from the former for subsequent analysis, except for individuals ktg001 (for which only non-UDG data were generated) and iam004 (for which both treated and untreated data were merged and processed as non-UDG treated for downstream analysis). We used a modified version of FilterUniqSAMCons_cc.py^57^ to ensure random choice of bases to collapse reads with identical start and end positions into a consensus, thereby removing PCR duplicates. Reads shorter than 35 bp and more than 10% mismatches to the human reference genome were filtered out.

### Contamination, sex determination, uniparental markers and kinship analyses

Sample contamination estimates were obtained using three different methods based on the mitochondrial genome^61^, on the X chromosome in males^62^ and on nuclear data^63^ (Supplementary Data 3). The ratio of coverage of X and Y chromosomes relative to autosomes was used to determine the biological sex of each individual^64^. We generated mitochondrial consensus sequences using SAMtools 1.5 mpileup and vcfutils.pl^60,65^. Base (BQ) and mapping quality (MAPQ) scores were set to MAPQ > 30 and BQ > 30, and only sites with at least threefold coverage were used. Haplogroups were assigned using Haplogrep 2.1.16 (ref. ^66^) and PhyloTree mtDNA tree Build 17 (18 February 2016)^67^ (Supplementary Data 4). For Y haplogroup inference we called SNPs from the International Society of Genetic Genealogy (http://isogg.org; v.11.110, 21 April 2016)) from bam files using SAMtools mpileup with option -B. We extracted sites with mapping and base quality greater than 30. Insertions and deletions, and sites showing multiple alleles, were excluded (Supplementary Data 5).

We ran kinship analysis with READ^68^ within each archaeological site (minimum of three individuals; Supplementary Fig. 2). When a pair of individuals with close kinship was found, such as first-degree relationships (parent–offspring or a full sibling), we excluded the individual with fewer SNPs covered from the analyses. This resulted in the removal from the analysis of iam4 (same individual as iam5), keb8 (same individual as keb1), iam6 (first-degree relative to iam004)^6^ and TAF012 (first-degree relative to TAF011)^10^.

### Population genomics analysis of pseudohaploid data

Data from over 300 ancient Eurasian, North African and Sub-Saharan African individuals, organized according to geography and chronology (Supplementary Data 2), were downloaded, mapped and processed though the same pipeline as used for newly generated data. The full ancient DNA dataset was merged with publicly accessible modern-day individuals sampled across the globe from the Simons Genome Diversity Project (SGDP) dataset^34^ for a 2.2 million SNP panel^64^. Alleles were sampled from bam files by randomly drawing one read with MAPQ > 30 and BQ > 30 per SNP site for each ancient individual (using SAMtools v.1.5.0 mpileup with option -B), and that position was treated as (pseudo)haploid. For non-UDG-treated data (ktg001) or merged UDG and non-UDG data (iam004) we trimmed off 10 bp of sequence-ends to avoid integration of miscoding C-to-T and G-to-A substitutions. For the published partial UDG-treated data (UDG-half), 2 bp were trimmed off the sequence-ends. SNPs showing more than two alleles were excluded from the data, leaving 1,379,466 SNPs for analysis.

Principal component analysis was performed using smartpca v.10210 (ref. ^69^). Principal components were calculated based on individuals from 18 Mediterranean Eurasian or North African modern-day populations from SGDP. Ancient individuals were projected onto the PCA space with options shrinkmode: YES and lsqproject: YES. An unsupervised model-based clustering algorithm, implemented in ADMIXTURE v,1.3.0 (ref. ^70^), was performed for *K* = 3–5 (30 runs) on a fully pseudohaploidized, linkage disequilibrium-pruned dataset of modern-day and ancient individuals from Mediterranean Eurasian or North African populations, leaving 812,092 SNPs for analysis. The results were parsed, aligned and plotted with pong^71^.

Popstats^72^ was used to calculate *f-*statistics^73^, with Mbuti set as the outgroup (Supplementary Data 6 and 7). Outgroup-*f*_3_ statistics were computed with the option –f3vanilla. Standard errors (SEs) were calculated with a weighted block jack-knife approach.

Admixture modelling was performed with qpAdm^74^ using ADMIXTOOLS v.5.0, through an adapted version of qpAdm_wrapper (https://github.com/pontussk/qpAdm_wrapper) that cycles through all possible subsets of the list of source populations provided (selected based on previous results), to test one-, two-, three- and four-way admixture models. SEs were computed with 5cM block jack-knife. We used a set of 11 reference populations whose power to disentangle divergent strains of ancestry present in Europe, North Africa and the Near East has previously been described and that are differently related to the sources tested^10,31,75^. Distantly related sources were explored and, where possible, also more proximate groups (geographically, chronologically or according to standing archaeological evidence). We tried to find the most parsimonious models consistent with the data (*P* > 0.05) by checking the lowest possible number of ancestry sources necessary to explain the ancestry in each test population (Supplementary Data 8–13). The Admixture event in KTG was dated using ALDER^76^ and DATES^77^ (Supplementary Information 8). We calculated conditional nucleotide diversity^78^ by estimation of the average number of mismatches between two individuals of the same population. SEs were estimated using a block jack-knife approach and a block size of 2,000 SNPs (Supplementary Fig. 9).

### Population genomics analysis of diploid data

Diploid genotype calls for a panel of 49,791,572 SNPs were performed for northwestern African ancient individuals with at least ninefold genome coverage (oub002, ktg004 and skh001), as well as relevant, previously published ancient individuals with sequenced high-coverage genomes. Before genotype calling, base quality in read ends was reduced and indel realignment conducted with GATK 3.5.0. Diploid genotypes were called using dbSNP v.142 as known SNPs, with GATK’s UnifiedGenotyper^79^. We computed average sequencing depth (avg.DP) over all called positions for each individual and filtered for QUAL > 30 and a depth span from fivefold to 3× avg.DP per individual, using BCFtools view. This dataset was merged with data from modern-day individuals from the SGDP dataset.

Individual heterozygosity was calculated from the number of variable positions divided by that of sequenced SNPs, using the –het command in PLINK 1.9 (ref. ^80^). We estimated the length and number of runs of homozygosity after filtering with the command PLINK –geno 0. MSMC^81^ input files were generated from VCF files. Filters for MAPQ > 30, minimum genotype quality of 50 and sequencing depth were used. Sites not passing these filters were masked out per individual. MSMC 0.1.0 was then run for each individual.

### Ethics and inclusion statement

The sampling for this study emerged from archaeology projects that involved local universities and researchers, including Y.B., whose involvement in research design included the selection of archaeological material for analyses as well as sampling supervision. The local relevance of this research is tied to the region’s history, and it is locally relevant in regard to describing the human past in northwestern Africa. The study was undertaken with the highest standards of archaeogenomic research, and relevant research by local scholars was cited.

### Reporting summary

Further information on research design is available in the Nature Portfolio Reporting Summary linked to this article.

## Online content

Any methods, additional references, Nature Portfolio reporting summaries, source data, extended data, supplementary information, acknowledgements, peer review information; details of author contributions and competing interests; and statements of data and code availability are available at 10.1038/s41586-023-06166-6.

## Supplementary information

Supplementary Information A description of the northwestern African archaeological sites studied and a detailed description of the methods used for analyses, supplementary results and supplementary discussions for these results.

Reporting Summary

Supplementary Data Supplementary Data 1–15.

## Data Availability

The sequence data generated for this study are available from the European Nucleotide Archive under accession no. PRJEB59008.
