# Supplementary material for: Northwest African Neolithic initiated by migrants from Iberia and Levant
Source: Nature. 2023 Jun 7;618(7965):550–6. doi: 10.1038/s41586-023-06166-6 (PMC10266975; doi:10.1038/s41586-023-06166-6)
Supplement: Supplementary file 1 — A description of the northwestern African archaeological sites studied and a detailed description of the methods used for analyses, supplementary results and supplementary discussions for these results. [file 41586_2023_6166_MOESM1_ESM.pdf]

nature portfolio

https://doi.org/10.1038/s41586-023-06166-6

Supplementary information

Northwest African Neolithic initiated by migrants from Iberia and Levant

In the format provided by the authors and unedited

SUPPLEMENTARY INFORMATION
Northwest African Neolithic initiated by migrants from Iberia and Levant

Luciana G. Simões[1], Torsten Günther[1], Rafael M. Martínez-Sánchez[2], Juan Carlos Vera-Rodríguez[3], Eneko Iriarte[4], Ricardo Rodríguez-Varela[5,6], Youssef Bokbot[7], Cristina Valdiosera[4,8], and Mattias Jakobsson[1,9]

[1]Human Evolution, Department of Organismal Biology and SciLifeLab, Uppsala University, Sweden
[2]Departamento de Historia, Universidad de Córdoba, Córdoba, Spain
[3]Área de Prehistoria, Departamento de Historia, Geografía y Antropología, Centro de Investigación en Patrimonio Histórico, Cultural y Natural, Facultad de Humanidades, Universidad de Huelva, Spain
[4]Universidad de Burgos, Departamento de Historia, Geografía y Comunicaciones, Burgos, Spain
[5]Centre for Palaeogenetics, Stockholm, Sweden
[6]Department of Archaeology and Classical Studies, Stockholm University, Stockholm, Sweden
[7]Institut National des Sciences de l'Archéologie et du Patrimoine, 6828 Rabat, Morocco
[8]La Trobe University, Department of History and Archaeology, Melbourne, Australia
[9]Palaeo-Research Institute, University of Johannesburg, P.O. Box 524, Auckland Park, 2006, South Africa

List of Supplementary Data Files

Supplementary Data File 1 . . . . Summary of the northwestern African archaeological samples in study

Supplementary Data File 2 . . . . List of ancient individuals used as comparative data

Supplementary Data File 3 . . . . Results from three different contamination estimation approaches

Supplementary Data File 4 . . . . Mitochondrial haplogroup determination with Haplogrep

Supplementary Data File 5 . . . . .Y chromossome haplogroup determination for the male individuals in study

Supplementary Data File 6 . . . . outgroup-$f3$ statistics

Supplementary Data File 7. . . . . $f4$-statistics

Supplementary Data File 8. . . . . qpAdm modelling OUB/IAM

Supplementary Data File 9. . . . . qpAdm modelling KTG

Supplementary Data File 10. . . . . qpAdm modelling KTG earlier subset

Supplementary Data File 11. . . . . qpAdm modelling KTG later subset

Supplementary Data File 12. . . . . qpAdm modelling SKH

Supplementary Data File 13. . . . . qpAdm modelling KEB

Supplementary Data File 14. . . . . Phenotypic Analysis using Hirisplex-S

Supplementary Data File 15. . . . . Library metrics information

Contents

SI 1. Archaeological context of studied sites **2**
Taforalt . . . 2
Ifri Ouberrid cave . . . 3
Kaf Taht el Ghar . . . 3
Ifri n'Amr ou Moussa . . . 3
Skhirat-Rouazi Necropolis . . . 4
Kehf-el-Baroud Cave . . . 4
Broader archaeological evidence of the onset of neolithic lifestyles in northwestern Africa . . . 4
Bones and teeth sampling . . . 5
SI 2. Radiocarbon dating **5**
SI 3. Ancient DNA extraction, library preparation and sequencing **5**
SI 4. Bioinformatics data processing and authentication **6**
SI 5. Sample contamination estimates **8**
SI 6. Molecular sexing of ancient individuals **8**
SI 7. Uniparental markers **8**
Mitochondrial haplogroup determination . . . 8
Y haplogroup determination . . . 9
SI 8. Pseudo-haploid population genomics analysis **9**
Kinship analysis . . . 10
Principal Component Analysis . . . 10
ADMIXTURE analyses . . . 10
f-statistics . . . 10
qpAdm admixture modeling . . . 14
OUB and IAM admixture modelling . . . 15
KTG admixture modelling . . . 15
SKH admixture modelling . . . 15
KEB admixture modelling . . . 16
Admixture graphs . . . 16
Conditional Nucleotide Diversity . . . 16
Admixture dating . . . 20
SI 9. Population genomics analysis on diploid calls **20**
Heterozygosity . . . 21
Runs of Homozygosity . . . 21
MSMC . . . 21
Phenotypic analysis . . . 21
Supplementary Information References **21**

SI 1. Archaeological context of studied sites

Taforalt

This cave, also known as "Grotte des Pigeons", is located in eastern Morocco (Beni-Snassen Mountains), close to the border with Algeria. As one of the major Palaeolithic sites in Morocco, the archaeological sequence covers the Aterian (Middle Stone Age, MSA) and Iberomaurusian (Later Stone Age, LSA). It was excavated in the 1940s by Ruhlmann and later, in the 1960s and 1970s, by Roche, who documented two adjacent necropolis areas with numerous Iberomaurusian burials. During the most recent excavations, conducted between 2003 and 2017[1], a series of closely spaced and inter-cutting primary burials were found in sector 10. In addition, sectors located on the south side of the cave (Sector 8) and at the front of the cave (Sector 9) provided overall relevant information on the layout of Iberomaurusian technology and social organization. Seven human bone samples from Sector 10 were directly radiocarbon dated with 95.4% probability to between 15,086 cal BP and 14,046 cal BP[1–3] and nine were included in a genomic study[1].

Ifri Ouberrid cave

The Ifri Ouberrid site is located in the village of Aïn Elleuh, about 15 km south of the town of Azrou, in the central part of the Middle Atlas, and is composed of two caves. The main cave was excavated in 2005 and provides a stratigraphy consisting of seven stratigraphic units representing two levels of prehistoric occupation[4]. The most recent (Niveau III) is attributed to the Early Neolithic, with some examples of impressed pottery with rocker motifs ("flammes pivotantes"). The dates obtained, on wood charcoal, are older for an acceptable Neolithic age in the region. These are KN-5920, 6839 ± 127 and KN-5919, 6846 ± 56, (both 7935-7580 cal BP, 94.5% BP probability). An earlier Epipalaeolithic level (Niveau IV), with important quantities of stone artifacts[5], has also been dated on charcoal, KN-5921, 7524 ± 105, 8544-8040 cal BP, 95.4% probability.

In this study we have directly radiocarbon dated an isolated phalanx, from the secondary cave, lacking a reliable archaeological context and potentially corresponding to a dismantled secondary deposit. This date is consistent with those obtained from the Niveau III of the main cave.

- oub002: IO.05/B, IO.05.F21 Enl.5. Phalanx. 6703±31 BP (Ua-56649), 95.4% probability 7660-7506 cal BP.

Kaf Taht el Ghar

Kaf that el Ghar is a large cavity located in the Jebal Gharghez, between the villages of Mechrouha and Yarrhite, next to the Mediterranean coast and the mouth of the Martil River. Miquel Tarradell, the first archaeologist to study the site, identified four main archaeological levels in the main room. Level III was a properly Neolithic horizon characterised by a very dark and soft deposit containing charcoal, fauna, bladelets, bone tools and many potsherds, notably cardial and channelled ware[6,7]. Further investigations were conducted between 1984 and 1987. Subsequently, between 1989 and 1994, a larger area (about 100 m^2) was excavated by a Franco-Moroccan team[8]. Unlike Tarradell's work, these campaigns revealed a much more complex stratigraphy with up to 42 units and a chronology extending from historic times to the Upper Palaeolithic[9]. The period attributed to the Early Neolithic was problematic from the outset. Its initial phase was derived from a charcoal dating to the 9th millennium cal BP in a layer with incised pottery[10], due to bioturbations resulting from mixed sediments and palimpsests[11,12]. The presence of cardial potsheds linked to cereal remains dated to 7286 ±94 cal BP (Ly-971 OXA, 6350 ± 85 BP)[13] refined the chronology of this first phase. In February 2012, within the framework of the AGRIWESTMED project, a limited intervention at KTG gave priority to the stratigraphic analysis of the preserved profiles and the flotation of soil samples. This work confirms the great potential of the Epipalaeolithic and Upper Pleistocene strata, as well as the remaining Neolithic strata. A careful analysis of the 30 cm thick Early Neolithic layer succession reveals the abundance of cultivated cereals (*Triticum dicoccum, T. monococcum/dicoccum, T. aestivum/durum, Hordeum vulgare*)[14] and domestic mammals (*Ovis aries*), which proved to be the oldest domestic taxa dated in the North-West Maghreb. In fact, this phase is clearly distinguished from the thick lower layers of the Epipalaeolithic by a very hard carbonate horizon devoid of pottery. Above the Early Neolithic layers, in turn, there are several units containing artefacts characteristic of the Middle Neolithic, such as pottery shards analogous to Ashakar ware (Gilman 1975). The material culture associated with this early Neolithic phase includes numerous decorated, impressed, incised, modeled, and channeled ware sherds[15]. These include cardial decoration in the form of simple impressions, rocker, and dragged motifs. The ceramic forms contain vessels with everted rims, constricted necks, and rounded or conical bases with lug handles. However, the use of red slip is not attested. This first phase is dated to between 7500 and 7000 cal BP from the radiocarbon dates obtained, mainly from domesticated and human remains[14,16].

We analysed the following four individuals from this site:

- ktg001: KTG'2012 Corte 26 A-B Ampliación, UE 1103 1ª Alz (well contextualized Early Neolithic level). Upper incisor tooth. 6410 ±30 BP (Beta-424637), 95.4% probability 7423-7267 cal BP.
- ktg004: KTG89, H30,3. Right mandible (fragment), infant. 6143±33 BP (Ua-56454), 95.4% probability 7159-6945 cal BP.
- ktg005: KTG89, I30, 1-3. Incisor tooth. 6461±34 BP (Ua-58118), 95.4% probability 7429-7285 cal BP.
- ktg006: KTG94, I27, 1019, maxilla fragment with 2 teeth (tooth sampled). 6206±33 BP (Ua-56455), 95.4% probability 7247-6995 cal BP.

Ifri n'Amr ou Moussa

About 90 km east of Rabat, near to the town of Khemisset, is placed the cave of Ifri n'Amr ou Moussa, on the Zemmour Plateau in the Oued Beth Basin. This site was previously known for the findings of bell beaker pottery[17] and some prehistoric burials[18], considered initially to be Chalcolithic, but now Neolithic. The site comprises 10 published radiocarbon dates on human bone, making it the best-dated Neolithic necropolis in Morocco so far. These dates correspond to burials 1-7, with a time range of 7316-6679 cal BP (95.4% probability)[19,20]. The cave has also yielded a Neolithic and Epipalaeolithic sequence

explored by a Moroccan-Spanish team in 2013 and later, by a Franco-Moroccan team[21]. Although not yet fully studied, the sequence comprises Neolithic layers marked by ash and charcoal deposits, contemporary with the burials. Those levels include some examples of cardial-like pottery impressed with simple and rocker motifs made with shells. Furthermore, although this Neolithic phase contains cereal remains, it lacks bones that can be confidently classified as domestic fauna. Wild fruit and cereal grain (Trench 2, SU 2006 and 2007) yielded dates (Beta-411102 [charred fruit] and OxA-34042 [*Hordeum vulgare*]) points to the arrival of cultivated cereals in this region ca. 7050 cal BP[22].

We analysed one individual from this site:

- iam004: IAM06.S1. Petrous bone. Beta-443596, 5970 ± 30 BP, 95.4% probability 6894-6679 cal BP[19].

Skhirat-Rouazi Necropolis

The cemetery of Skhirat-Rouazi was placed next to the Atlantic shore, 30 km southwest Rabat. Associated with a shell midden and excavated between 1982 and 1984, the site has over one hundred primary burials, many of them children. A rich collection of grave goods was preserved, such as 132 pottery vessels, ivory bracelets, and ostrich eggshells beds[23–26]. Pottery, as MN ceramics from Magarat el-Khil, shares characteristics of Saharan type, as flat bases and flower pot or globular-shaped vessels with straight or inverted rims, internal tubular handles, and impressed comb and cord (à la cordelette) decors combined with colored slip[11,27]. Although its chronology has been debated, being linked to more recent times[28], seven C14 samples on human bone place the use of this cemetery throughout the 7th-millennium cal BP[20], 95.4% probability 6733-6121 cal BP.

We analysed three individuals from this site:

- skh001: SKHIRAT.S40. Tooth. 5574±35 BP (Ua-56836), 95.4% probability 6437-6295 cal BP.
- skh002: SKHIRAT.S46. Molar. 5818±33 BP (Ua-58120), 95.4% probability 6733-6500 cal BP.
- skh003: SKHIRAT.S100. Molar. 5425±35 BP (Ua-56837), 95.4% probability 6298-6121 cal BP.

Kehf-el-Baroud Cave

The cave is located in the municipality of Ziaïda, 10 km north-east of the town of Ben Slimane, 50 km east of Casablanca. It is opened in a reef limestone massif and is the result of an underground hydrographic network, consisting of two interconnected cavities. Known since the middle of the last century, when it was excavated by C. Plessis, and between 1969 and 1972 by A. De Wailly[29]. The most recent excavations were carried out between 1991 and 1997 by INSAP researchers[30,31], documenting occupations between the Epipalaeolithic and Bronze Age, including Bell Beaker pottery, cooper tools and evidence of ivory craftmanship. One of the main phases corresponds to a Middle-Late Neolithic, suggested by undecorate pottery, flint tools and domestic animals. There are five known C14 dates published today for the Neolithic phase, on bone and worked ivory. Although those made in the 1970s on bone showed a high standard deviation[29], they do not differ greatly from those published later[31]. The range of these dates is 6395-5596 cal BP (95.4% probability). The human individual previously analysed archaeogenetically[19], provided the sample Beta-443603, 4940 ± 30, 5728-5596 cal BP 95.4% probability.

Broader archaeological evidence of the onset of Neolithic lifestyles in northwestern Africa

In Mediterranean Iberia, the new economic and technological structures linked to the Neolithic seem to have made a strong appearance after 7500 cal BP. Rain-fed cereals and annual legumes together with domestic livestock such as sheep, goats, cows, and pigs are present from the outset, following an archaeological hiatus related to the last Mesolithic hunter-gatherers. Impressed pottery is already present at that time, being abundant impressed-groove, Almagra red-slip type decorations and incised mostly from 7400 cal BP onwards[32]. The oldest radiocarbon dates on short-lived domestic samples (sheep, cereals, etc.) from Mediterranean Iberia are from Peña Larga (Álava), Mas d´Is (Alicante) and Guixeres de Vilobi (Penedes)[33].

The site of Kaf Taht el Ghar holds the oldest directly dated evidence of domesticates in northwestern Maghreb. Other sites with Cardial pottery have yielded slightly more recent dates (such as Magharat el Khil) or no longer accepted dates, following bioturbation issues (such as Kef Boussaria). In Ifri Oudadane, no domestic animals in the Early Neolithic A (ENA) phase have been found. The ENA phase of Ifri Oudadane is in fact highly contentious[15,34–36]. The roots of the debate lie on the radiocarbon date of a charred lentil of controversial taxa. This single date (Ifri Oudadane: Early Neolithic A, Beta-295779, 6740 ± 50 BP) would imply an older age for the Eastern Rif Early Neolithic (95.4% probability, 7679-7510 cal BP). Other studies challenging this single evidence for an earlier component of Neolithic lifestyle in northwestern Africa argue that the oldest records of non-controversial domestic items in the Eastern Rif (Ifri Oudadane site) date from around 7100 cal BP (Early Neolithic A, *Pisum sativum*; Beta-341129, 6160 ± 30, 7160-6959 cal BP 95.4% probability; Early Neolithic B, *Triticum* sp., Beta-318608, 6140 ± 30, 7159-6946 cal BP 95.4% probability)[14,37].

While the Neolithisation process in today's Morocco has been understudied, evidence of ceramics with a Saharan-like appearance have been found at sites such as Hassi Ouenzga[38] and Adrar n'Metgourine[39]. The origin and development of pottery

in the Saharan territory is well established[40]. Furthermore, the appearance in the archaeological record of Saharan pottery styles in western Morocco associated with pastoralism throughout the 7th millennium cal BP has also been documented[39]. However, beyond ceramics, the material evidence of the lifeways of these groups in present-day Morocco is not well known, considering a lack of sites during this period in areas such as the Eastern Rif[41]. To this day, only Atlantic coastal sites highly dependent on sea resources such as Magharat el Khil (Tangier), and cemeteries, such as those of El Kiffen and Skhirat (Tangier-Casablanca), have provided contextual information[39].

The pottery of the necropolis of Skhirat-Rouazi is well known and its technological features, such as funicular handles, red slip, and corded decoration (cord-wrapped roulette), have been directly related to the so-called Ashakar Ware. Ashakar ware, known from the Tangier area, has an analogous chronology, mid-7th millennium cal BP, at the site of Magharat el Khil, caves B and C. Some of the formal features of Ashakar and Skhirat ceramics have a Saharan origin that is thought to have spread from the interior of the continent from at least the mid-7th millennium cal BP. Environmental fluctuations and dry episodes in the Sahara could have promoted population movements and the progressive spread westward of African bovine pastoralism[39]. In Magharat el Khil, phases related to this pottery and chronology show the full adoption of agriculture (cereals and pulses)[14], as well as domestic fauna. The coastal character of this site determines a palaeo-biological ensemble type shellmidden, where coastal resources are dominant[39].

Bones and teeth sampling

No fieldwork was required for this study since the archaeological material used to perform the research was already curated at the Institut National des Sciences de l´Archèologie et du Patrimoine (INSAP) in Rabat, Morocco. A scientific cooperation agreement between INSAP and La Trobe and Uppsala Universities was signed prior to sampling, which was performed under the supervision of Prof. Youssef Bokbot at INSAP. Samples were taken in a portable glove bag using previously sterilised equipment at INSAP. Drilling samples into bone powder was sidestepped to avoid contamination. Instead, complete bone/teeth elements were taken in sterilised tubes/bags to the ancient DNA laboratory for further cleaning and sampling. Diamond cutting disks were used to cut 1 cm^2 of petrous bone if this was still attached to the temporal bone.

SI 2. Radiocarbon dating

All individuals were directly radiocarbon dated using accelerator mass spectrometry (AMS) at the Tandem Laboratory at Ångström, Uppsala, except for ktg001, which was dated at the Beta Analytic Carbon dating laboratory and iam004, which was obtained from[19].

Bone samples were pre-treated and dated according to the following steps: (1) The surface is mechanically cleaned (scraping, in some cases sand blasting). (2) The sample is ultrasonically cleaned in boiled, distilled water (pH 3). (3) Grinding in mortar. (4) 0.8 M HCl is added, stirred (30 minutes, circa 10 °C) (apatite removed). Soluble fraction is referred to as fraction A. (5) Distilled water kept at pH 3 is added to the insoluble fraction, which is heated while stirring (6-8 hours, 90 °C). The insoluble part is referred to as fraction C and soluble part is referred to as fraction D. Fraction D should give the most relevant age, since it contains most of the organic parts (the "collagen”) of the original bone. The fraction to be 14C-dated is combusted to CO2 which is graphitised using a Fe-catalyst reaction prior to the accelerator determination. Fraction D has been dated. Radiocarbon dating was performed on the same bone piece/element as ancient DNA (aDNA) extraction to avoid spurious dates (e.g. radiocarbon analysis performed on maxilla and DNA extracted from tooth in maxilla). Radiocarbon calibration was performed using Oxcal v4.4 and the IntCal20 dataset[42].

SI 3. Ancient DNA extraction, library preparation and sequencing

Teeth and bone powder, or if feasible depending on the sampled bone element, bone pieces, were sampled in a dedicated clean room at Uppsala University, Sweden. Prior to sampling, we conducted a series of stringent procedures that aimed at minimizing surface contamination. All surfaces of bones and teeth were first exposed to UV-radiation (6 J/cm2 at 254 nm) followed by mechanical cleaning of surfaces removing leftover dirt. Samples surfaces were then wiped with bleach (0.5% solution), wiped again with Millipore water and finally re-exposed to UV-radiation. For teeth samples, we targeted pieces of root tips[43]. Bone or tooth pieces and/or powder were then subdivided into ca. 50 mg per vial. During sampling, particular care was taken at preserving relevant morphological characters of bones and teeth, minimizing the impact of the procedure on the samples.

DNA extraction, library building and amplification were performed in dedicated clean rooms at Uppsala University, Sweden. Thirty to sixty milligrams of bone were used for DNA extraction following[44], with adaptations as in[45] and an initial pre-digestion step with 1mL of 0.5M EDTA pH=8 for 30 minutes at 37°C[46]. Pre-digestion buffer was discarded. Sample digestion was performed overnight with 1mL of the following buffer: 0.5M EDTA pH=8, 1M Urea, 100 µg/mL Proteinase K. Finally, DNA was eluted in 110uL of EB buffer. For all extractions, two negative controls were included per batch.

For each sample, we started by using 20 uL of non-UDG (uracil-DNA-glycosylase) treated DNA extract (e1) to build a double-stranded DNA library. P5 and P7 adapters were ligated to blunt-end repaired DNA as described in[47]. These libraries were used to assess DNA quality and quantity by estimating endogenous DNA content, damage patterns (i.e C to T deamination patterns) and fragment size typical of ancient DNA[48] in order to validate its authenticity.

Samples with $>5\%$ endogenous human content were considered for furthered data generation. In order to maximize the use of SNPs (transitions and transversions) for genomic analyses, we used the remaining DNA extract to build four to six double-stranded DNA libraries which were subjected to UDG treatment to remove ancient DNA damage (by excision of Uracils with USER from BioNordika) as in[49].

For all samples except ktg001 and iam004 we generated a second extract (e2) and for most samples we also generated a third extract (e3) following[50] with adaptation of the binding buffer, which was prepared by adding 9 ml sodium acetate (5M) and 1.25 ml sodium chloride (5M) to 500mL of Qiagen PB buffer; acetic acid was added to adjust pH (pH=4-5)[51]. These extracts were eluted in 80 uL of EB. Damage-repaired libraries were built as described above using 15-20 uL of DNA extract. A list of all extracts and libraries can be found in Table S1.

Table S1. List of extractions and libraries performed for the ancient samples.

Sample	Adapted Yang extractions	Adapted Dabney extractions	Blunt-end libraries	Damage-repair libraries
oub002	2	0	1	6
ktg001	1	0	5	0
ktg004	2	1	1	15
ktg005	2	1	1	11
ktg006	2	1	1	11
iam004	3	0	3	4
skh001	2	1	1	10
skh002	2	1	1	9
skh003	2	1	1	7

Two negative controls were included in all library batches. All blanks were carried along the processing steps and screened on qPCR, which was also used to determine the optimal number of PCR cycles for library amplification as described in[49]. None of the blanks showed DNA content comparable to that of any sample and therefore were not amplified with PCR. Each library was amplified twice, following conditions described in[49] in four amplification reactions of 25uL, or alternatively in two 50uL reactions. Between 12 and 20 PCR cycles were used, depending on qPCR Cq indication. Each single PCR was tagged with a unique 7 bp indexed primer[47]. Amplifications of the same library (with the same indexing primer) were pooled and purified with AMPure XP beads (Agencourt; Beckman Coulter, Brea, CA). Library quality was checked by electrophoresis on Tapestation (Agilent High Sensitivity D1000 ScreenTape, Agilent Technologies, Cary, NC) and DNA concentration was quantified using Qubit dsDNA HS (High Sensitivity) Assay Kit (Invitrogen).

Initial screening sequencing runs were performed on ca. twenty amplified and purified libraries, pooled in equimolar concentrations. Pools were sequenced on Illumina HiSeq X at the SNP & SEQ Technology Platform in Uppsala. To reach higher coverage, four to 10 libraries were pooled equimolarly and sequenced to depletion (ca. 50% clonality after merging library data), depending on proportion of human DNA, clonality and genomic coverage.

SI 4. Bioinformatics data processing and authentication

Data was demultiplexed according to the indexed primer sequence and processed with MergeReadsFastQ_cc.py[52] or Adapter Removal v2.1.7[53]. Illumina adapters were clipped followed by merging of forward and reverse paired-end reads when an overlap of at least 11 bp was found. Single-ended merged reads were mapped against the human reference genome build 37 (hs37d5) using bwa aln 0.7.13[54] with the non-default parameters: -l 16500 (disabling seeds) -n 0.01 (higher than default fraction of missing alignments) -o 2 (maximum of 2 gap opens)[55,56]. We verified the authenticity of our data by observing the characteristic damage patterns of cytosine deamination at fragments' ends[48]. A high frequency (>15%) of C to T (5') and G to A (3') deamination was observed in all non-UDG libraries.

For each library, we merged bam files resulting from all re-sequencing rounds using samtools merge v1.5[54]. We estimated contamination for each single library using mitochondrial DNA (mtDNA) data[57], to avoid merging data from libraries with potentially higher levels of contamination when compared to other libraries for the same individual. Estimates are based on

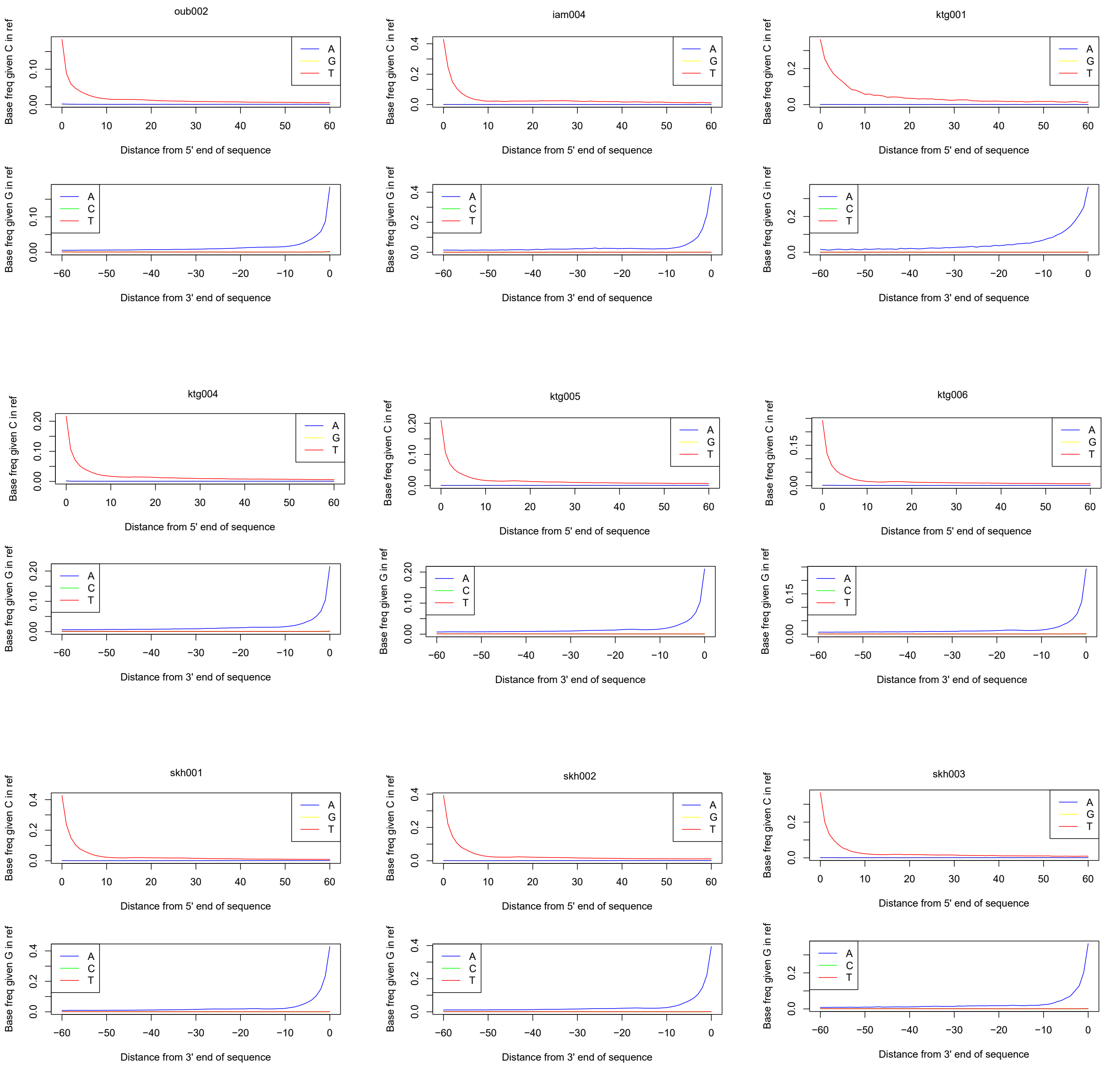

Supplementary Figure 1. Deamination plots computed from each of the analysed individuals' initially screened non-UDG library.

contradicting signals in the mtDNA data, by identifying private or near-private (<5% in 311 modern mtDNAs) consensus alleles in each ancient individual. The analysis was restricted to positions where coverage was at least 10-fold and base qualities >30. Since no substantial differences were found between contamination estimates of libraries of the same individual, we proceeded to merge all data generated for each individual. To take advantage of UDG treated data and the possibility of using all SNPs in genomic analysis, we merged treated and untreated data separately and use preferably only damage repaired data for subsequent analysis. For ktg001, only non-UDG data was generated. For iam004, data generated from UDG-treated libraries alone was insufficient for confident genomic analyses. Thus, we merged UDG treated and untreated data and for further analyses we processed it as non-UDG. We used a modified version of FilterUniqSAMCons_cc.py[52] to ensure random choice of bases to collapse reads with identical start and end positions into a consensus, and thus removing PCR duplicates. Reads shorter than 35 bp and more than 10% mismatches to the human reference genome were filtered out.

SI 5. Sample contamination estimates

Sample contamination estimates were performed using three different methods. Firstly, contamination was estimated based on phylogenetically informative sites on the mitochondrial genome using ContamMix[58], which uses a probabilistic model and using Markov chain Monte Carlo to estimate the proportion of contamination. We then calculated contamination based on X chromosome in males, who carry a single X chromosome and thus contamination is reflected in higher mismatch rates for polymorphic and adjacent to polymorphic sites[59]. As a first step, a binary count file for the X chromosome was generated per individual using ANGSD v.0.902, discarding low quality bases (-minMapQ 30 -minQ 30). Differences between polymorphic sites and adjacent sites are tested with Fisher's exact test. We considered further results whenever p-value <0.05. A maximum likelihood framework is then applied to compute the estimates and standard errors are calculated with jackknife. We report results for test 1, which samples all reads from each site, producing a more precise and sensitive estimate compared to method 2, which randomly samples one read per site. Finally, we used verifyBamID v.1.1.2[60] to estimate nuclear contamination. This method uses a mathematical model that relates observed sequence reads to an hypothetical true genotype, to check whether reads in a BAM file match previously known genotypes for a group of individuals and whether the reads are contaminated as a mixture of two samples. It has been previously used for ancient samples[49,61]. We used the 1000 genomes reference panel vcf file (ALL.wgs.phase3_shapeit2_mvncall_integrated_v5b.20130502.sites.vcf.gz) and report results outputted in the FREEMIX column, which indicates the proportion of non-reference bases observed.

The three different methods used for estimating contamination were inconsistent (Supplementary Data File 3). However, most of the analyses in this study are based on autosomal data, therefore we relied on the two methods used to estimate nuclear contamination.

SI 6. Molecular sexing of ancient individuals

Two methods were used for determining the biological sex of each individual. The first one considered the ratio of sequences aligning to the X and Y chromosomes[62] and the second considered the ratio of coverage of the X chromosome relative to the autosomes (X-rate) and the Y-chromosome relative to the autosomes (Y-rate)[63]. While results were generally consistent across methods, we report the second method estimates. Also, we did not observe any evident deviations from an equal ratio of males and females amongst the analysed sites.

SI 7. Uniparental markers

Mitochondrial haplogroup determination

We generated mitochondrial consensus sequences using samtools 1.5 mpileup and vcfutils.pl[64,65]. Base and mapping quality scores were set to a minimum of 30 and only SNPs with at least 3-fold coverage were used. Haplogroups were assigned using Haplogrep 2.1.16[66] and PhyloTree mtDNA tree Build 17 (18 Feb 2016)[67].

The average coverage for the mtDNA genomes was 956.98x. The polymorphisms found are identified in Supplementary Data File 4. We find haplogroups U6 and/or M1, that have been associated to the Back to Africa migration event and that became prevalent in North Africa before the Holocene[68–70] in all analysed sites. Haplogroup U6, previously reported in Upper Palaeolithic Taforalt[1] and Early Neolithic IAM[19] is found from the Epipalaeolithic to the Middle Neolithic in our samples (oub002, ktg001, iam004, skh003).

The mt haplogroup composition in KTG is notable, as it is representative of the complex admixture patterns found in this population. While ktg001 shows a typical North African U6 haplogroup, ktg004 and ktg006 have mt haplogroups associated to the European Neolithic (HV0 and J1c3j), and previously found across Europe from the Neolithic[71,72] and in Bell Beaker and Bronze Age Sardinia[73], respectively. Ktg005 shows mt haplogroup U5, which has been widely found in WHG. In particular, the haplogroup U5b2b1a has been recently reported in Mesolithic individuals from Sicily[74].

In SKH, the pastoralism associated gene flow into northwestern Africa is observable in the mt haplogroup composition. The mt haplogroup assigned to skh002 (J2a2d) has previously been observed in Chalcolithic Israel[75], the closest proxy to the source population of the pastoralism associated migration into East Africa, while M1a1b (skh001) has been previously reported in the pastoral Neolithic population of present-day Kenya[76].

Table S2. MtDNA haplogroup determined for the northwestern African individuals in this study.

Individual	mtDNA coverage	Overall Haplogrep Rank	mt Haplogroup	haplogroup previous observations
oub002	2853.42	0.9437	U6a6b	Upper Palaeolithic Morocco
ktg001	1110.31	0.8740	U6	Present-day North Africa
ktg004	2819.18	1.0000	HV0+195	Neolithic Europe (e.g. Southern France LBR,PEN)
ktg005	988.414	1.0222	U5b2b1a	Mesolithic Sicily/Ajvide PWC
ktg006	253.991	0.9963	J1c3j	Neolithic Central European/ Bronze Age Sardinia
iam004	8.92969	1.5426	U6a7	Neolithic Morocco
skh001	492.879	0.9500	M1a1b	Pastoral Neolithic Kenya
skh002	64.9684	0.9128	J2a2d	Chalcolithic Israel
skh003	20.6917	1.7304	U6c	Present-day North Africa

Y haplogroup determination

For Y haplogroup inference, we called SNPs from the International Society of Genetic Genealogy (ISOGG; http://isogg.org (version 11.110, April 21, 2016)) from bamfiles using samtools mpileup -B. We extracted sites with mapping and base quality of at least 30. Insertions and deletions and sites which displayed multiple alleles were excluded. For all male individuals except ktg001 (for which we use damage repaired and unrepaired data and therefore limited this analysis to transversions, leaving no derived alleles to establish the Y haplogroup), we kept transition sites (with cautious interpretation) because for damage repaired data, these are unlikely to result from aDNA damage. We report derived states for each individual as well as key ancestral alleles within the haplogroup, to verify the certainty of the call. Additionally, we double checked that no ancestral alleles upstream of the final haplogroup contradicted the call. All individuals displayed scattered inconsistent derived states for markers of other Y chromosome haplogroups. However, we refuted those observations after guaranteeing that such derived alleles were preceded by ancestral upstream alleles. When several haplogroup defining markers display derived allele states, we report only three of them (Supplementary Data File 5).

We observe a demarcated Y-haplogroup structure. We could determine the Y-haplogroup for two out of the three males of the EN site of KTG and both carried G2a2b2a1a1c1a. Haplogroup G2a2 has been previously associated with European farmers, and it has been identified in Neolithic Anatolia, Balkans, Hungary and Iberia[77–79]. The two males of the MN site of SKH carried Y-haplogroup T1a1a, which has been previously identified in most of the male individuals from Chalcolithic Israel[75]. Haplogroup T has also been observed in the LN northwestern African site KEB and in Neolithic samples from Jordan[19,80].

Table S3. Y chromosome haplogroup determined for the northwestern African individuals in this study.

Individual	Y haplogroup	Teminal SNPs	haplogroup previous observations
ktg001	nd	nd	nd
ktg004	G2a2b2a1a1c1a	CTS4472, CTS6763	Neolithic Europe
ktg006	G2a2b2a1a1c1a	Z3435	Neolithic Europe
skh002	T1a1a	L905	Chalcolithic Israel
skh003	T1a1a	PF5620	Chalcolithic Israel

Our uniparental markers analysis indicates that the maternally inherited mtDNA lineages within Neolithic sites with admixed ancestry (KTG and SKH) are represented proportionally to the corresponding ancestry fractions found in each of these groups, i.e. the observed mt lineages are associated with the ancestry backgrounds identified after population genomics analyses, including those present in lower proportions. This implies that females from both admixing groups were involved in the KTG and SKH admixture events. The observed Y-chromosome haplogroups, on the other hand, exclusively trace back to the incoming populations (European farmer for EN KTG and Eastern pastoralist for MN SKH) contributing to the admixture. This could indicate a biased (and higher) contribution from male farmers into KTG and male pastoralists into SKH, as the Y chromosome lineage identified in TAF and IAM (E1b1b)[1,19] is not observed in these populations. However, the low sample sizes do not allow for a confident assessment.

SI 8. Pseudo-haploid population genomics analysis

After quality control assessment, we compiled a dataset comprising the data generated in this study as well as a panel of over 300 ancient Eurasian, North African and Sub-Saharan African individuals from 60 populations relevant for the analyses

(Supplementary Data File 2,[1,19,49,56,63,72–103]), organized according to geography and chronology. Genetic data from all comparative ancient individuals was mapped and processed though the same pipeline (described in section SI 4) as the new data in this study, in order to homogenize data treatment. The compiled aDNA dataset was merged with publicly accessible worldwide modern individuals from the Simons Genome Diversity Project (SGDP) datasets[104]. However, since most of the ancient samples in the comparative dataset were enriched for a targeted set of SNPs, we limited the pseudo-haploid analyses to the 2.2M SNP set[63]. Alleles were sampled from bamfiles by randomly drawing one read with MAPQ>30 and BQ>30 for each SNP site for each ancient individual (using samtools v 1.5.0, mpileup –B), and that position was assumed to be homozygous for the sampled allele. For non UDG damage-repaired data (ktg001 and iam004), or merged UDG and non-UDG data (iam004), we trimmed 10 bp off of sequence ends, where deamination damage typically accumulates, to avoid integrating miscoding C to T and G to A substitutions. For published partial damage repair data (UDG-half), 2 bp were trimmed off of the reads ends. SNPs showing more than two alleles were excluded from the data, leaving 1,379,466 SNPs for analysis.

Kinship analysis

We looked for kin-relationship between individuals of the same site. We ran READ[105] within each archaeological site, for which we had data for at least 3 individuals. This tool uses a normalized pairwise genetic distance between ancient individuals to infer their relatedness. READ predictions of familial relationships are conservative and reliable even when a low number of overlapping SNPs is available, which is ideal to infer kin-related connections from ancient DNA data. When close kinship was found, such as first-degree relationships (parent-offspring or a full sibling), we excluded one individual of the pair from the analysis. The criteria followed was lower coverage and less covered SNPs than the related individual which was kept in the analysis.

We removed iam4 (same individual as or twin of iam5), keb8 (same individual or twin of keb1)[19], as well as TAF012 (within the confidence intervals of first degree relative to TAF011)[1] from the analyses.

We found that iam004 was a first degree relative to iam6 published in[19]. We kept iam004 in the analyses and excluded iam6.

Second-degree family links are represented by half-siblings, grandparent-grandchild, aunt/uncle-niece/nephew, or double cousins. In spite of the indications of small population sizes in all analysed groups, we did not find any further kin relationships.

Principal Component Analysis

We performed Principal Component Analysis (PCA) to characterize the genetic affinities of our Moroccan genomes with other modern and ancient populations. Principal components were calculated on 18 Mediterranean Eurasian and North African modern populations from SGDP panel using smartpca v.10210[106] with outliermode: 2. Ancient individuals were projected onto the PCA space with options shrinkmode: YES and lsqproject: YES. PCA plots were generated using R 3.4.0[107] and the ggplot2 package[108].

ADMIXTURE analyses

We ran an unsupervised model-based clustering algorithm, implemented in ADMIXTURE v1.3.0[109]. The analysed data set was fully pseudo-haploidized by randomly selecting one allele at heterozygous sites for modern individuals. Data was LD pruned using PLINK v1.90b4.9[110] with parameters –indep-pairwise 200 25 0.4, leaving 812,092 SNPs for analysis. We ran ADMIXTURE with different random seeds from K=3 to K=5 and 30 replicates for each K. We refrained from interpreting higher K because the results were not consistent, as every solution was supported by very few runs. We present results parsed, aligned and plotted with pong[111].

We observe that at K=3, components maximized in sub-Saharan Africa (grey), Neolithic Anatolia (beige) and WHG (yellow) are represented. At K=4, the ancient northwestern Africa ancestry differentiates (depicted in brown). Pre-Neolithic individuals as well as EN IAM are exclusively represented by that component and later ancient northwestern Africans consistently maintain a fraction of the ancestry represented by that brown component. It is observable in ancient East Africans from the Pastoral Neolithic period and in modern North African populations. It is also represented in ancient Levantine populations, which may depict shared drift. At K=5 (portrayed in the main text), a western component differentiates (in green), maximized in modern Levantine populations (Bedouin B and Yemenite) and in some individuals from Levant Neolithic and Chalcolithic). It is observable in Pastoral Neolithic East Africa and in northwestern African it is introduced from the MN, in the site of SKH, and maintained in later periods, including in the Canary Islands Guanches.

f-statistics

Popstats[112] was used to calculate all *f*-statistics[113]. Outgroup-*f3* statistics with the option –f3vanilla was computed to estimate shared genetic drift between northwestern Africa populations, new and previously published, and other ancient groups. Standard errors are calculated with weighted block jackknife. The full outgroup-*f3* results, with the entire set of ancient populations analysed, including sub-Saharan African, can be found in Supplementary Data File 6.

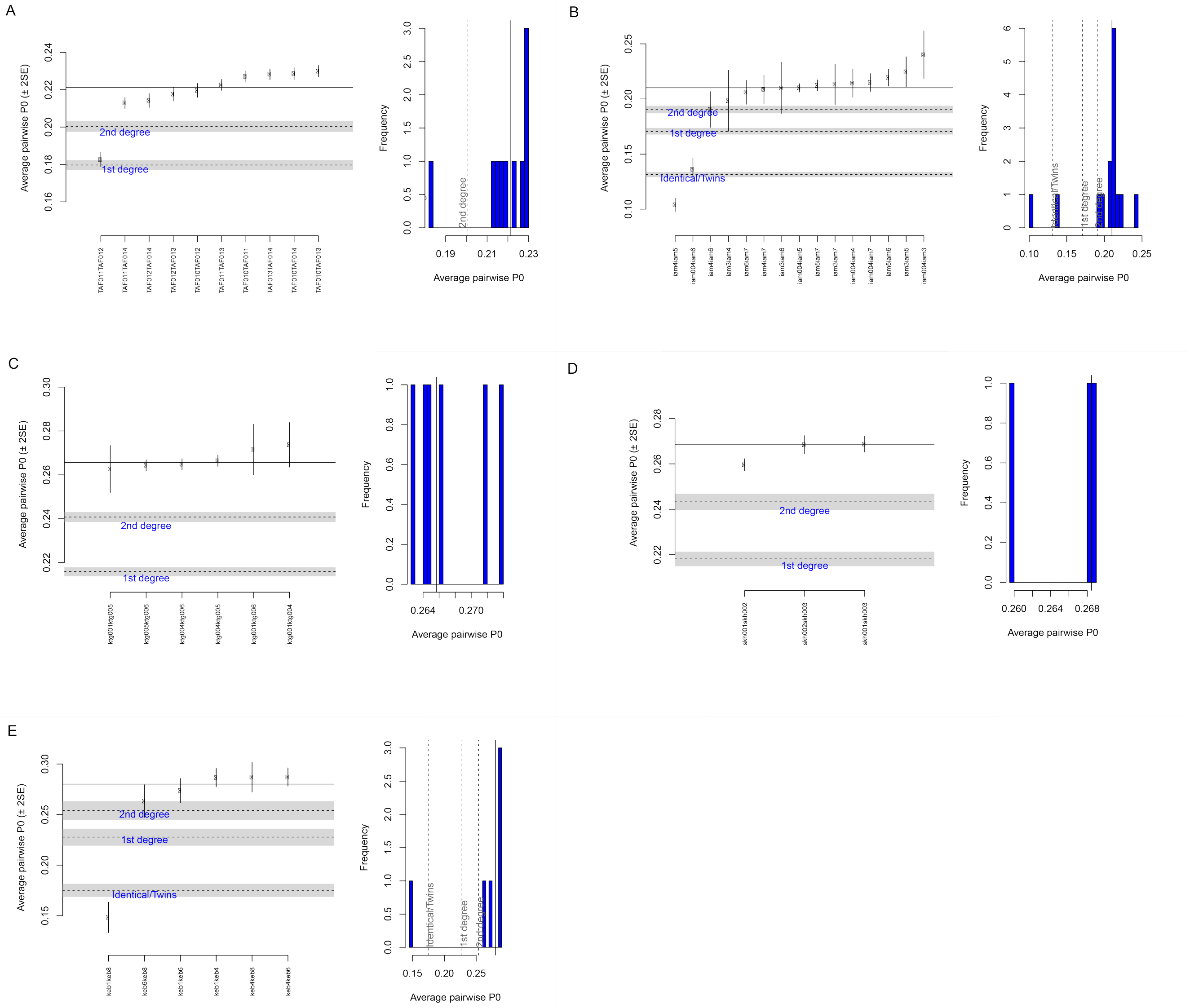

Supplementary Figure 2. READ's average pairwise P0 values for A. Taforalt (TAF); B. Ifri n'Amr ou Moussa (IAM); C. Kaf Taht el Ghar (KTG); D. Skhirat (SKH); E. Kelif el Boroud (KEB).

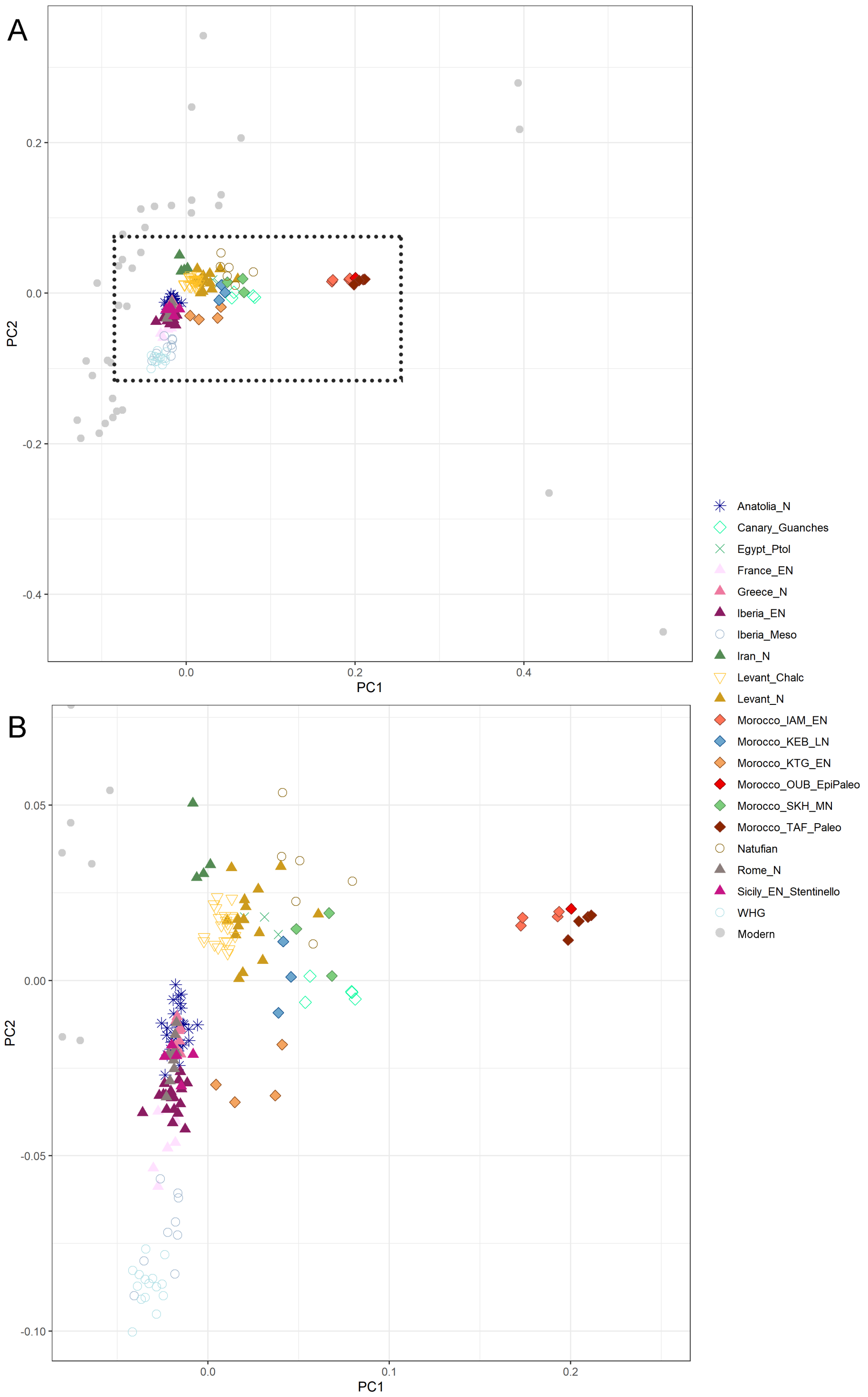

Supplementary Figure 3. A. Principal Component analysis, on 2.2M SNPs dataset, with projection of ancient individuals onto Principal Component axes defined by 18 Mediterranean Eurasian and North African modern populations of the SGDP panel (grey dots). B. Zoom into the projected ancient individuals. The full set of individuals under each legend item is listed in Supp. Table 2.

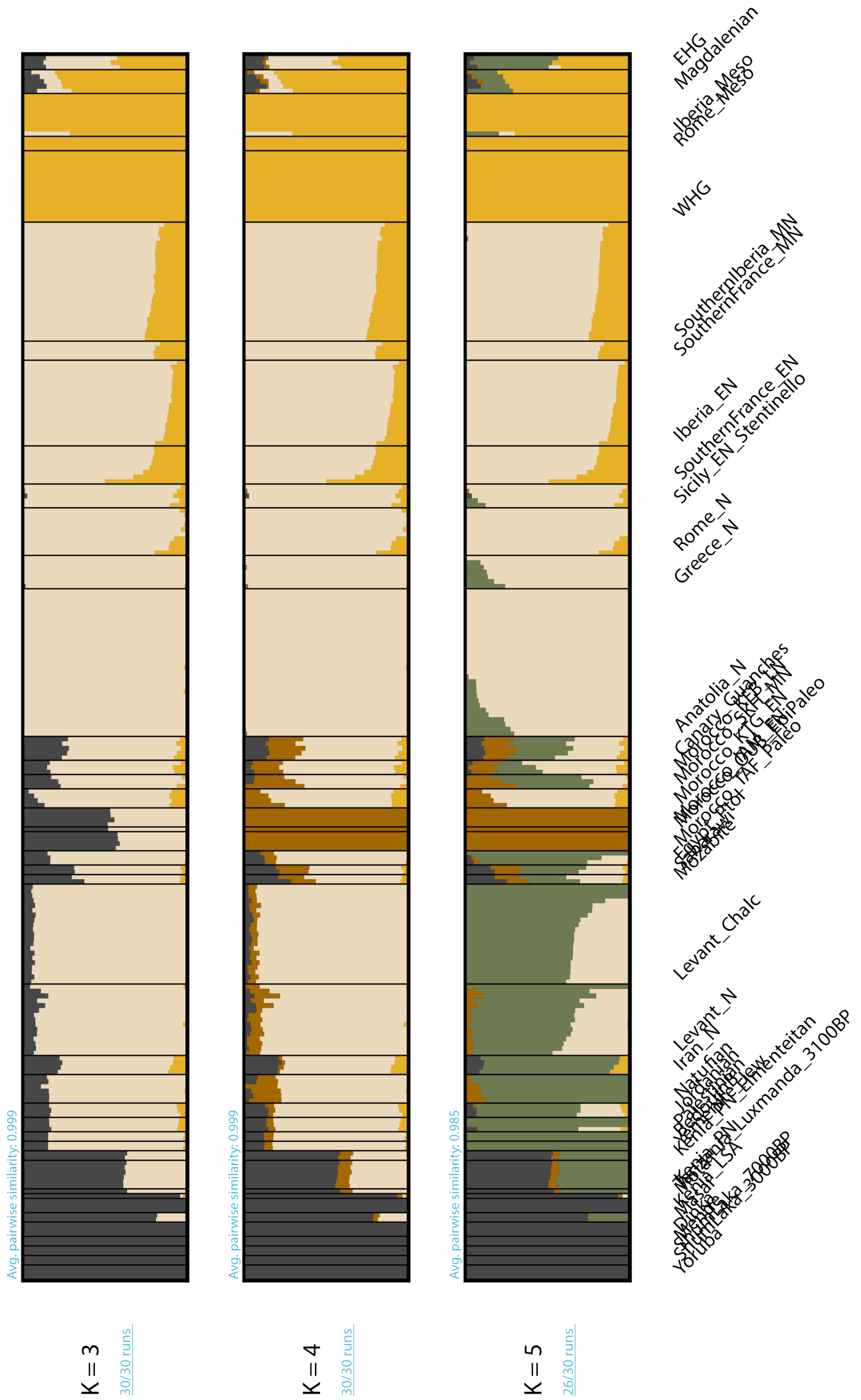

Supplementary Figure 4. ADMIXTURE analysis with 2.2M SNPs dataset for K=3 to K=5. Each horizontal bar represents one individual and color fractions depict the fraction of the given individual's ancestry coming from each cluster.

Outgroup-*f3* results show a marked tight cluster of genetic similarity between autochthonous northwestern Africans, representative of the genetic continuity observable from the Late Pleistocene until the EN in that region. From the EN (KTG), that continuity is disrupted and more drift is shared with European Neolithic populations, specially Iberian, French, Sicilian and Sardinian. The same trend is obervable in LN KEB. However, a gap of overall lower outgroup-*f3* values is noticeable in MN SKH.

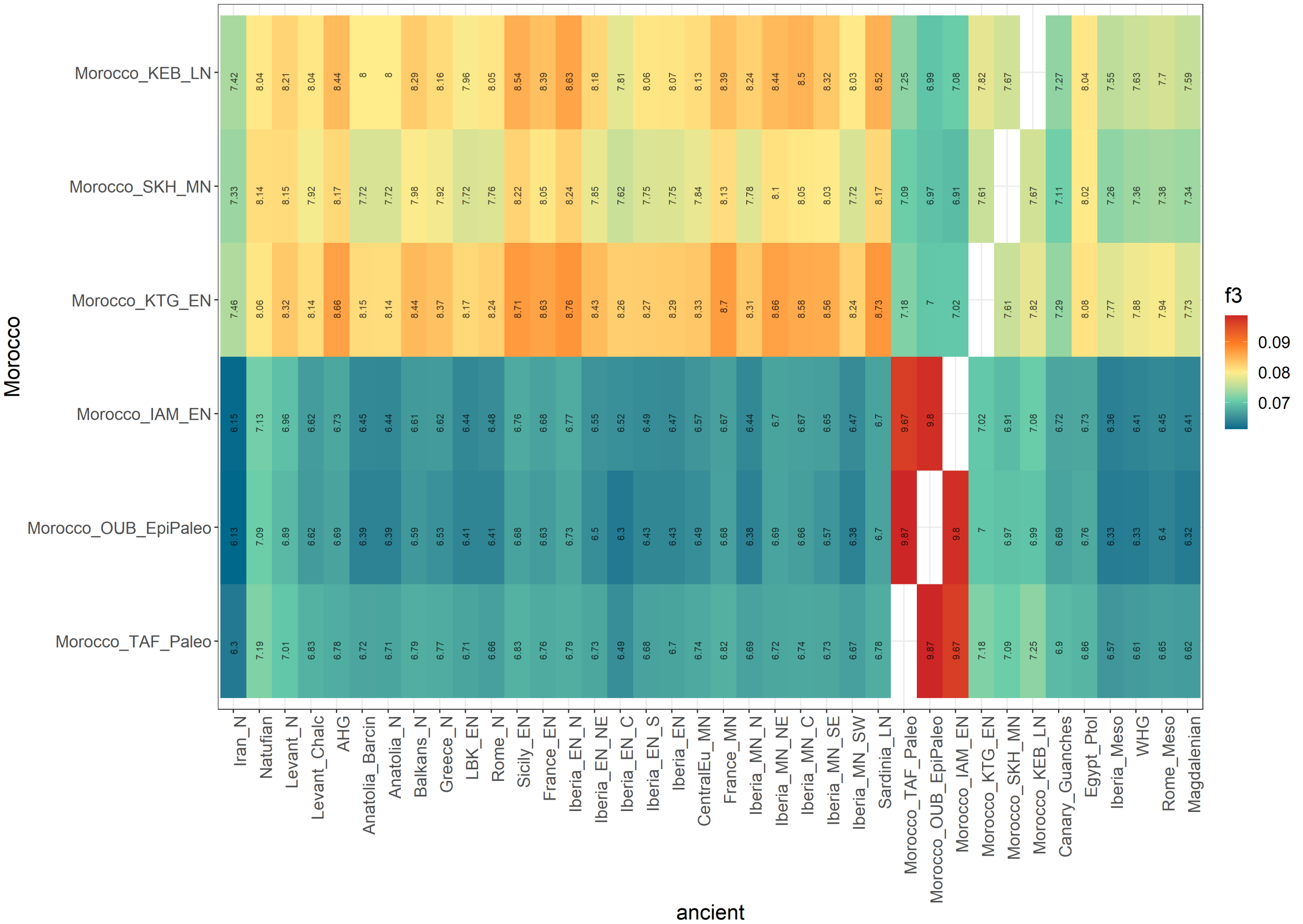

Supplementary Figure 5. Genetic similarity matrix, calculated as pairwise outgroup-*f3* statistics of the form *f3*(Mbuti; ancient northwestern African population, ancient test population).

qpAdm admixture modeling

Admixture modeling was performed with qpAdm_wrapper (https://github.com/pontussk/qpAdm_wrapper) and Admixtools v5.0[113,114]. qpAdm makes use of *f4*-statistics to model a test population as a mixture of proposed sources ("left" populations), verifying if these are linearly related to a set of reference populations ("right" populations). The resulting matrix is summarized into a single statistics to assess if the null model of admixture can be rejected, p-values are calculated using a likelihood ratio test comparing a constrained null model to an unconstrained alternative model. It estimates ancestry proportion coefficients and Standard Error (SE) with 5cM block jackknife. The original qpAdm wrapper cycles through all combinations of the set of reference populations as sources. We used an adapted version of the wrapper that cycles through all possible subsets of the provided list of source populations. This way, we tested 1-way, 2-way, 3-way and 4-way combinations of source populations. We used a set of 11 references combining populations whose power to disentangle divergent strains of ancestry present in Europe, North Africa and the Near East has been previously described and that are differently related to the sources tested[1,75,86,115]: Mbuti,Mota,Us_Ishim,MA1,GoyetQ116-1,Kostenki14,Han,Papuan,AHG,CHG,Natufian,Villabruna,Iran_N. We conducted our formal admixture testing with qpAdm based on the results obtained from the previous analyses. We thus started by exploring source populations that were more distantly related to the test populations, and that maximize the components that could explain their ancestry (according to the exploratory analyses). When possible, we also conducted modelling with more proximate

groups (geographically, chronologically or according to standing archaeological evidence) that are plausible sources for the ancestry in the tested populations.

We tried to find the most parsimonious models by checking the lowest possible number of sources (including 1-way models) necessary to explain the ancestry in each test population. If no 1-way model showed a good fit (p>0.05), we looked for plausible 2-way or 3-way. We also investigated 4-way models when the population history so required, as indicated by prior analysis. We report cases where adding an extra source improves the previously accepted model (by increasing the p-value) or when relevant for the analysis.

OUB and IAM admixture modelling

A 1-way model, using TAF as source was sufficient to explain the ancestry in OUB and IAM (Supplementary Data File 8). Interestingly, when a European Neolithic source is added, the model becomes unfeasible for OUB but not for IAM, that is then modelled as a misture of 95-96% TAF and 4-5% European Neolithic, with an increased p-value for Sicily EN.

KTG admixture modelling

We started by modelling KTG population with a set of distant sources: Anatolia Barcin, TAF, WHG, and GoyetQ2 or EHG. We use TAF as a northwestern African source and not IAM or OUB because IAM postdates KTG and thus could have been affected by events after the arrival of European farmers to modern-day Morocco (and consequent admixture event with local populations) while OUB is represented by a single individual.

While a 2-way model (TAF, Anatolia Barcin) does not fit the data (p<0.05), a 3-way model (TAF, Anatolia Barcin, WHG) is viable, showing that a WHG source is necessary to model KTG. In addition to the accepted 3-way model (Anatolia Barcin, TAF, WHG), we tested adding a fourth source: GoyetQ2 or EHG. Iberian farmers and Eastern Mediterranean farmers (including those from Sicily) have been shown to retain some Magdalenian- and EHG-related ancestry, respectively[74,86,88]. Thus, we leverage this difference in HG ancestry between different European Early farmer groups to validate the 2 source proximal models that indicated Iberia as the best fitting source. To investigate which fourth ancestry source could fit KTG farmers, we use a rotation strategy[88,116], rotating Goyet Q2 (Magdalenian associated) to the outgroups and added EHG as a source, and vice-versa. When rotating GoyetQ2 to the references and setting EHG as source, the model becomes infeasible. The opposite (Goyet-Q2 set as source and EHG rotated to the reference set) results in a feasible model. Taken together, these results add support to an Iberian origin of European farmer ancestry found in KTG.

We then modelled KTG using TAF and more proximal Mediterranean European Neolithic sources, which have been shown to combine Anatolian Neolithic with a smaller proportion of European HG ancestry. Feasible models feature Iberian groups or Sicily Stentinello as European early farmer sources, but the models which include Iberia EN groups rank with a 5- or 6-fold higher p-value, indicating that using Iberia as a source results in an improved model fit. Detailed results are shown in Supplementary Data File 9.

Given that the four KTG individuals can be divided into two chronological subsets - an earlier (ktg001 and ktg005) and a more recent (ktg004 and ktg006), we modelled these as described above for the whole group.

Overall, we obtain a two-fold higher proportion of TAF ancestry in the earlier KTG individuals than in the most recent (Supplementary Data Files 10 and 11). For the 2-way models, almost all populations tested can be fitted as a proxy for the European Early farmer ancestry in KTG earlier subset (p>0.05) with the interesting exception of farmers from Southern France. The best ranking models (with highest p-values) have Iberian EN groups as sources (Supplementary Data File 10).

For the more recent subset of KTG individuals, 2-way models including TAF and Iberian EN groups are clearly the best fit, particularly Central and Southern Iberian farmers (Supplementary Data File 11).

SKH admixture modelling

To model SKH, we used populations present in northwestern Africa before the MN as well as Iberia EN (the most likely migrating population during the EN) to investigate if the genetic pool present in the region during the EN was sufficient to model the population of SKH or if additional migration from Iberia during the MN was observable. Additionally, we used Levantine Neolithic/Chalcolithic and Iran Neolithic populations to account for the novel ancestry noted in SKH in the exploratory analyses.

The only model that fits the data is a 2-way model between TAF and Levant Chalcolithic. Adding Iberia EN or KTG does not improve the model fit, indicating that the contribution of the European EN gene pool to SKH was minor (Supplementary Data File 12).

We also experimented with the references set used in[76], that has been useful to detect Levantine ancestry in East African Pastoralist Neolithic groups. The results confirm that Levantine ancestry is needed to model SKH, as a simple admixture between pre-Neolithic northwestern Africans and Iberian farmers does not fit the data (Supplementary Data File 12).

KEB admixture modelling

The admixture modelling for KEB was not too conclusive because all of the models tested are accepted. This is likely due to a combined effect of the small sample size of the KEB population (n=3) and the extreme low coverage of all samples. It is expected that when the target population has a very reduced sample size, nonoptimal models are identified as plausible by qpAdm, because the power to reject wrong models is reduced[116].

Our modelling efforts seem to indicate that it is possible to model KEB as a mix of populations that were already present in Morocco during the Neolithic, such as KTG and SKH, or even as a direct descending population from those. Our interpretation is based on the most parsimonious solution that the LN population of KEB can be modelled with ancestry previously present in northwestern Africa, and not necessarily need the inclusion of major migration waves into the region after the MN (Supplementary Data File 13).

Admixture graphs

We used ADMIXTOOLS 2 findGraphs function[117] to systematically search for admixture graphs. This tool explores the best fitting admixture graph topology for a set of f-statistics in a rigorous and unsupervised way, instead of evaluating a single graph for a specific topology as is usually done with qpGraph, and thus avoiding crafting input graphs. We tested the fit of modelled graphs with different number of migrations (m), using the functions qpgraph_resample_multi() and compare_fits(). We defined Mbuti as the outgroup and set the following populations to be modelled: Levant Neolithic, Levant Chlcolithic, Anatolia Barcin, Iberia EN, TAF, KTG, SKH. The choice to include both Levant Neolithic and Chalcolithic is tied to the uncertainty of which Levantine population serves as the best proximal source of the Levnatine pastoralism-associated ancestry that has also been detected in East Africans[76], and in this study in ancient northwestern Africa. Models with one, two and three migration events were inspected.

The best fitting topology for m=1 (defined by the lowest likelihood) models KTG as a mixture of a pre-Iberia EN node and the northwestern African branch.

When a second migration event is allowed, we obtain a graph fitting the admixture event of KTG as well as modelling TAF (Morocco_Paleo) as admixture between a Levantine source (which has been described as related to Natufian HGs[1]) and an African branch.

The best admixture graph results with three migration events fits SKH as a mixture between a majority of a basal Levantine ancestry an ancestral African ancestry, with a long private branch, in addition to the two migration events previously described. The fit of basal lineages into SKH and the long branch probably try to account for this population's unique genetic composition with ancestry from different groups. Possible scenarios are that the genetic variation of SKH is rooted in (possibly several) different and not yet sampled sources, not represented in this model. Previous efforts to search for a possible source of the West Eurasian ancestry detected in East Africans have revealed no consensus regarding which sequenced ancient Levantine population serves as the best fitting source of this ancestry[75,80]. Similarly, here we observe that Levant Neolithic or Chalcolithic are likely not proximal sources of the Levantine ancestry in SKH. A possible scenario is that the admixture between such Levantine component and a North African component took place to the East of North Africa, either in an event common to that giving rise to East African pastoralist populations or not. None of the currently sampled ancient populations is the best single proxy for the source population of the North African related ancestry in these admixed groups. Future studies in northeastern Africa (e.g. present-day Egypt) will hopefully help unravel this process and provide a better understanding of the proximal sources involved in this admixture event.

The model fits of the best graphs with two and three migration events were significantly different (p=4.051075e-08), strengthening the addition of a third migration event, leading to SKH.

Conditional Nucleotide Diversity

We calculated Conditional Nucleotide Diversity as described in[55] to assess levels of genetic diversity in several ancient groups, including some for which there is only SNP capture data available. This method is based on estimating the average number of mismatches between two individuals of the same population. Within the available possibilities, pairs of individuals were selected considering the highest genomic coverage available for each group and contemporaneity. Standard errors were estimated using a blockjackknife approach and a block size of 2000 SNPs.

TAF and IAM display levels of genetic diversity that are considerably lower than any other of the analysed populations (Figure 9), in agreement with the very low diversity estimate obtained for OUB. The genetic diversity levels observed in EN KTG and MN SKH, while modest, are much higher than in those native northwestern African populations, comparable to the values observed in some other Neolithic Mediterranean populations, such as Anatolia Barcin Neolithic or Rome Neolithic. Another clear shift is evident between the MN and the LN in KEB, with an increased level of diversity in LN individuals, which could be indicative that by then northwestern African populations had recovered from an extended period of reduced population sizes.

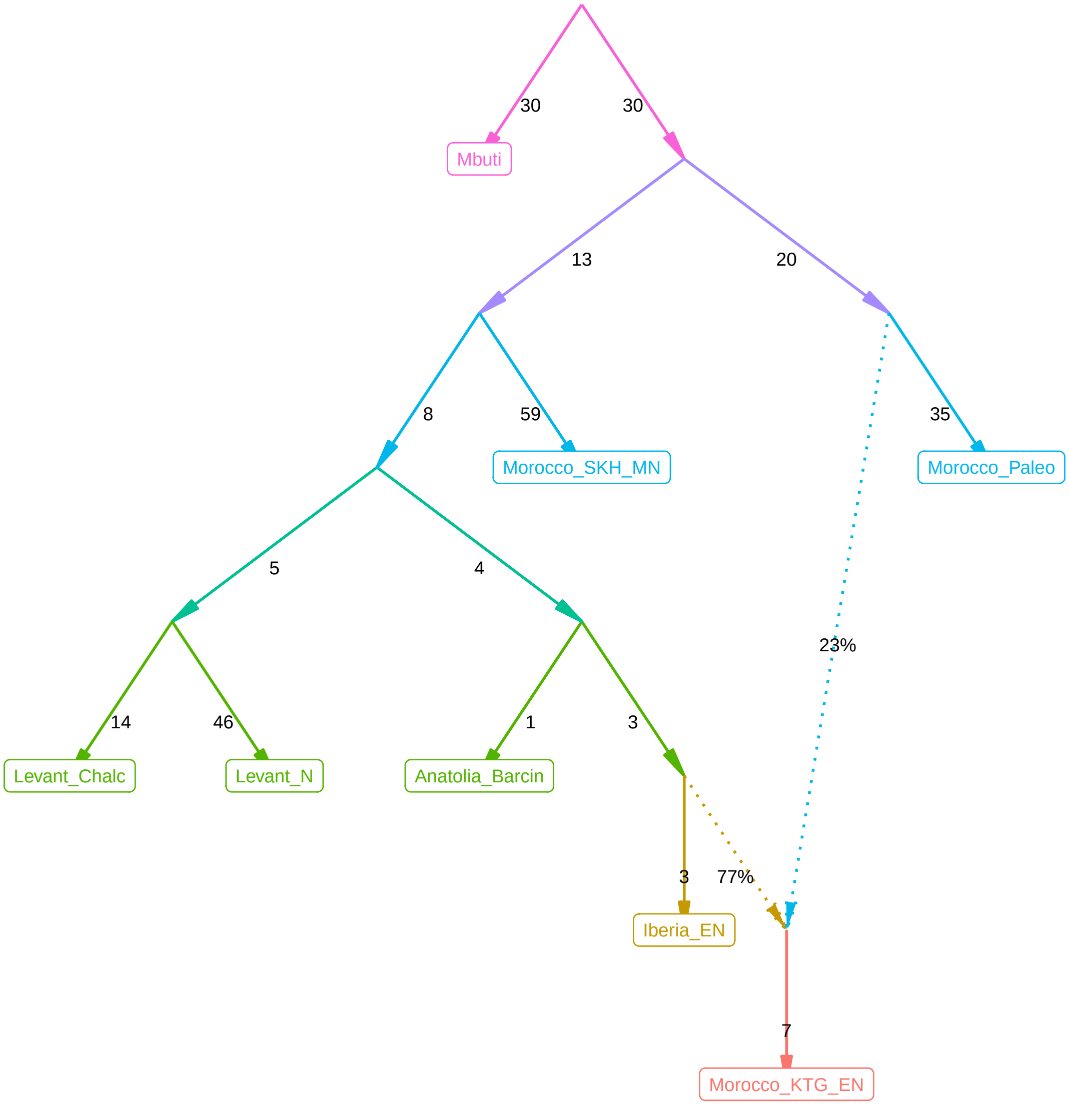

Supplementary Figure 6. Best admixture graph, with one migration event.

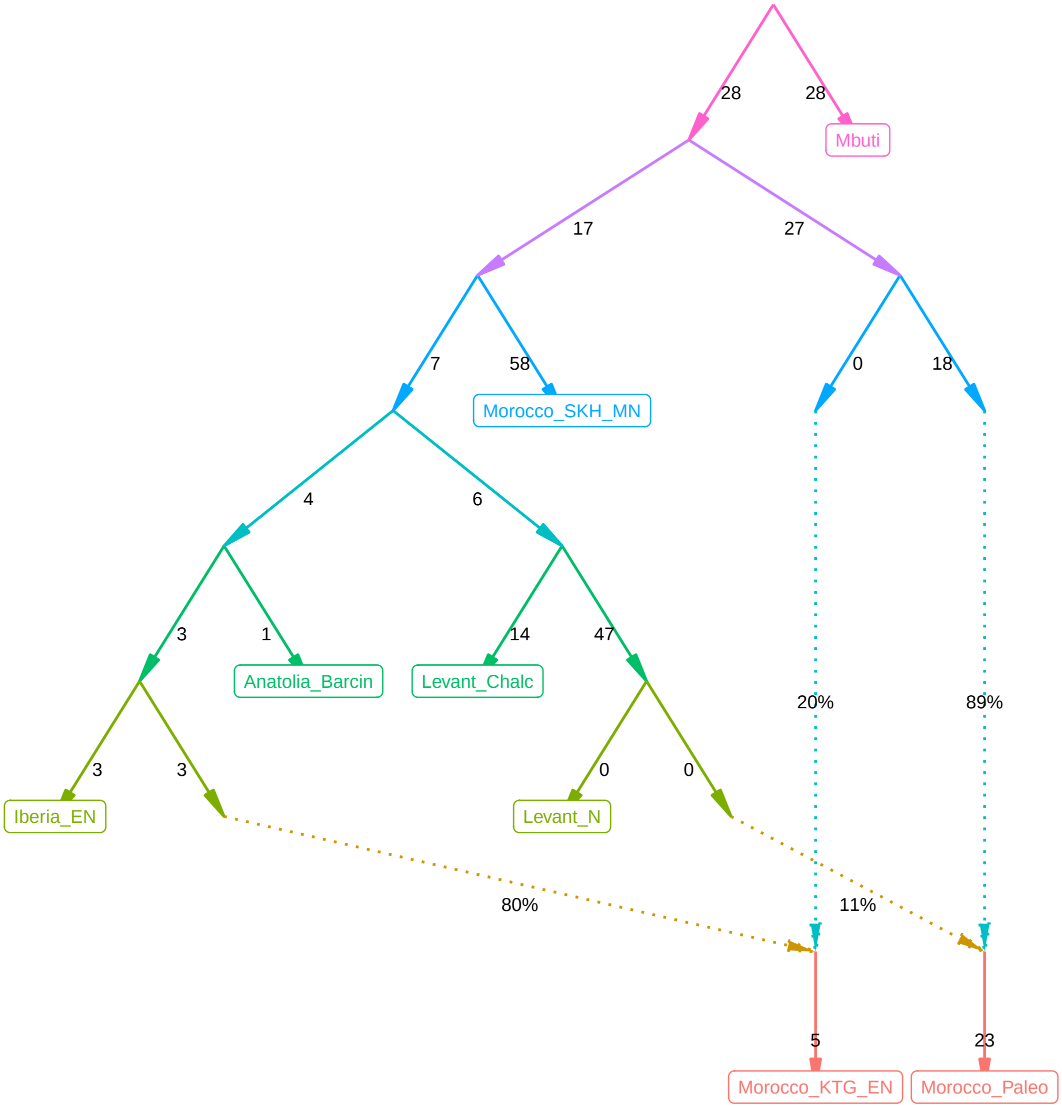

Supplementary Figure 7. Best admixture graph, with two migration events.

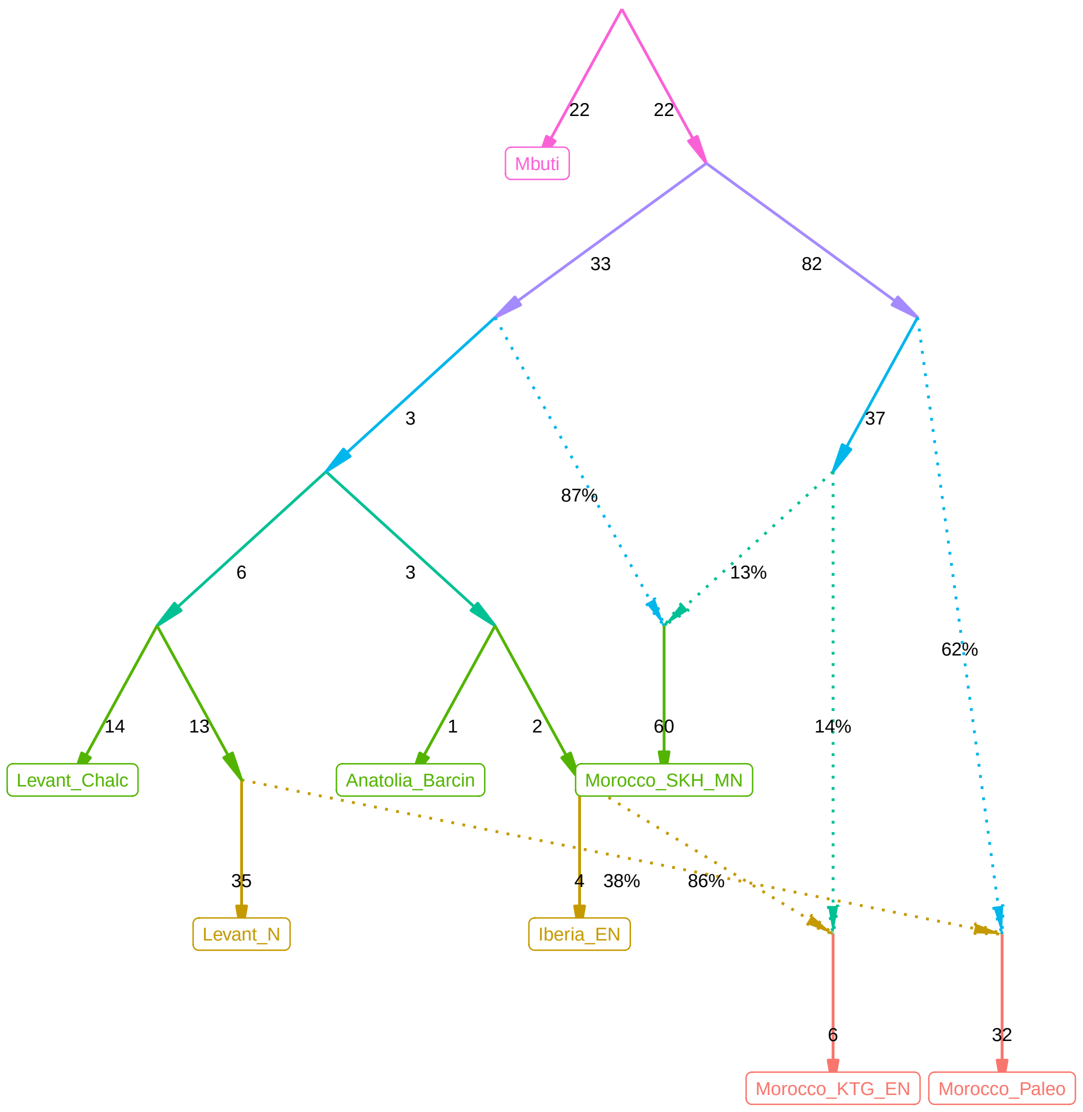

Supplementary Figure 8. Best admixture graph, with three migration events.

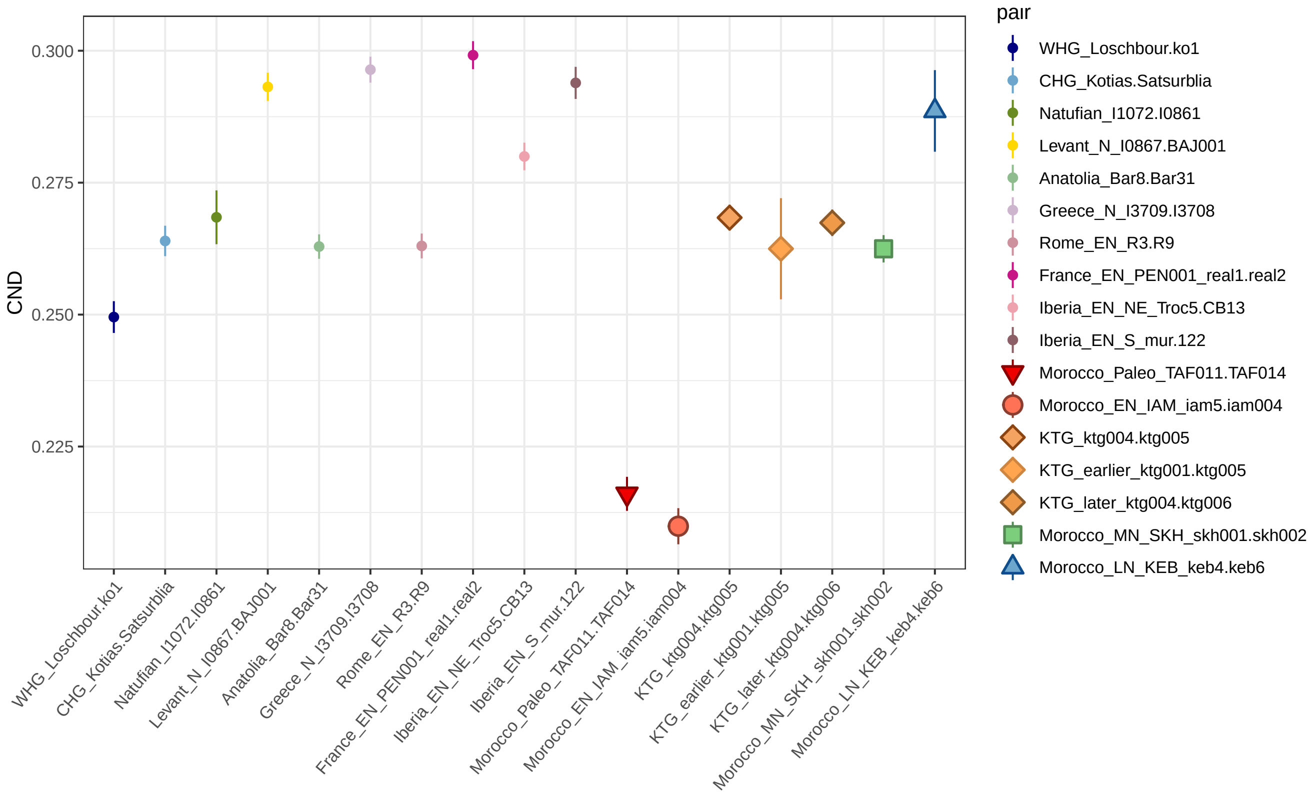

Supplementary Figure 9. Conditional nucleotide diversity (CND) calculated as the average pairwise mismatches between individuals. Individuals are grouped according to geographic and chronological proximity, or according to highest coverage if several individuals per population are available, which yield the highest number of SNPs for analysis. Western Hunter Gatherer (WHG), Caucasus Hunter Gatherer (CHG). Neolithic is denoted with N and EN Early Neolithic is denoted with EN. Note that the diversities calculated for CHG and WHG are overestimates, as the individuals representing CHG are separated by three millennia and those representing WHG are separated by >1,000 km. Standard Errors computed with a blockjackknife using 2000 SNPs. The error bars represent ±2 SE.

Admixture dating

We dated the admixture event of KTG using ALDER[118] and DATES[119]. ALDER uses a weighted linkage disequilibrium (LD) model to infer mixture proportions. We used an adapted script that allows computing of 1-ref weighted LD with only two individuals in the test population. DATES implements a regression-based ancestry covariance estimate that can be applied to single individuals. As source populations we used TAF and Iberia_EN or Sicily Stentinello.

Table S4. Admixture dating results estimated with ALDER.

Target	Source 1	Source 2	Test Result	2-ref decay
KTG	TAF	Iberia EN	Test FAILS (z=1.85, p=0.064)	
KTG	TAF	Sicily Stentinello	Test FAILS (z=0.58, p=0.56)	
KTG older	TAF	Iberia EN	Test FAILS (z=0.00, p=1)	
KTG older	TAF	Sicily Stentinello	Test FAILS (z=0.00, p=1)	
KTG recent	TAF	Iberia EN	Test SUCCEEDS (z=4.10, p=4.2e-05)	13.28 +/- 3.24
KTG recent	TAF	Sicily Stentinello	Test FAILS (z=1.34, p=0.18)	

SI 9. Population genomics analysis on diploid calls

Ancient individuals with genome coverage >9x generated in this study as well as relevant previously published ancient individuals with sequenced high coverage genomes (oub002, ktg004, skh001, baa001[99] – South African Stone Age HG, Mota[101] – East African Stone Age HG, Ust_Ishim[81] – Paleolithic from Siberia, Loschbour[56] – Western HG, Kotias[83] – Caucasus HG, sf12[49] – Scandinavian HG, Stuttgart LBK[56] – Early Neolithic from Germany and ne1[120] – Early Neolithic from Hungary) were

Table S5. Admixture dating results estimated with DATES.

Target	Source 1	Source 2	Test Result
KTG	TAF	Iberia EN	7.851 +/- 1.852
KTG	TAF	Sicily Stentinello	8.052 +/- 1.952
KTG older	TAF	Iberia EN	10.177 +/- 2.688
KTG older	TAF	Sicily Stentinello	10.333 +/- 2.840
KTG recent	TAF	Iberia EN	5.969 +/- 2.081
KTG recent	TAF	Sicily Stentinello	6.058 +/- 1.847

merged with the modern individuals of the SGDP dataset[104] after diploid genotype calling. The SNP panel consists of the variants found in publicly available individuals from the SGDP panel (https://reichdata.hms.harvard.edu/pub/datasets/sgdp/), provided in variant only vcfs, which were then converted into plink format, resulting in a total of 49,791,572 SNPs. For ancient data, we reduced the base quality of possible damages in read ends (Ts in the first five bp and As in the last). Indel realignment was conducted with GATK 3.5.0[54] using indels identified in phase 1 of the 1000 Genomes Project[121]. Diploid genotypes were called using using dbSNP version 142 as known SNPs, with GATK's UnifiedGenotyper and the parameters -stand_call_conf 50.0, -stand_emit_conf 50.0, -mbq 30, -contamination 0.02, and—output_mode EMIT_ALL_SITES. We computed average sequencing depth (avg.DP) over all called positions and filtered for QUAL>30 and a depth span from 5x to (3*avg.DP) x using bcftools view.

We used this dataset to compute additional measures of population diversity and demographic patterns (heterozygosity and runs of homozygosity).

Heterozygosity

Individual heterozygosity was calculated from the number of variable positions divided by number of sequenced SNPs, after LD pruning, using plink –het.

Runs of Homozygosity

Runs of homozygosity were analysed to investigate ancient North African populations' demographic histories. Large populations have few, short ROH, but isolated or bottlenecked populations have more and longer ROH. Admixed groups have the fewest ROH, whereas consanguinity leads to very long ROH[122]. We estimated length and number of RoH using Plink 1.9[110,123] after filtering with –geno 0 for ancient individuals as well as for modern SGDP individuals, for comparison with known populations RoH patterns. The following parameters were used –homozyg-density 50,–homozyg-gap 100,–homozyg-kb 500,–homozyg-snp 100,–homozyg-window-het 1,–homozyg-window-snp 100,–homozyg-window-threshold 0.05, and–homozyg-window-missing 20.

MSMC

MSMC[124] input files were generated from VCF files for relevant modern genomes from the SGDP dataset[104] as well as selected, high coverage ancient individuals. Filters for minimum mapping quality of 30, minimum genotype quality of 50 as well as sequencing depth filters (only accepting between half and double the average genome-wide sequencing depth) were employed. Sites not passing these filters were masked out per individual. MSMC[124] 0.1.0 was then run per individual with the parameters –fixedRecombination and -r 0.88.

Phenotypic analysis

We used the 24 SNPs used in HIrisplex-S[125] to predict eye, hair and skin color. ANGSD v. 0.933 was used to infer genotype likelihoods (GL) for the individuals with genome coverage >9x, with parameters minMapQ 30, minQ 30, setMinDepth 5, doCounts 1, doGlf 2, doMajorMinor 3. GL were then converted to genotypes, using a filter of 0.8. Whenever genotype assignment was dubious, we used mpileup to discern said genotype. HIrisPlex predictions can be applied on a worldwide scale and independently from bio-geographic ancestry knowledge[126]. These results should be interpreted with reservations, because it is not unlikely that ancient functional variants that are unknown today had a role in determining ancient populations' phenotypes, especially considering the complex genetic architecture of pigmentation traits[127,128].

We did not include the insertion polymorphism rs312262906 into the prediction model. The results are shown in Supplementary Data File 14.

Supplementary Information References

1. van de Loosdrecht, M. *et al.* Pleistocene north african genomes link near eastern and sub-saharan african human populations. *Science* **360**, 548–552 (2018).

2. Barton, R. *et al.* Origins of the iberomaurusian in nw africa: new ams radiocarbon dating of the middle and later stone age deposits at taforalt cave, morocco. *J. Hum. Evol.* **65**, 266–281 (2013).

3. Humphrey, L. T. *et al.* Earliest evidence for caries and exploitation of starchy plant foods in pleistocene hunter-gatherers from morocco. *Proc. Natl. Acad. Sci.* **111**, 954–959 (2014).

4. Mikdad, A., Nekkal, F., Nami, M., Zielhofer, C. & Amani, F. Recherches sur le peuplement humain et l'evolution paléoenvionnementale durant le pléistocène et l'holocène au moyen atlas central: Résultats préliminaires. *Bull. d'archéologie Marocaine* **22**, 53–71 (2012).

5. Hajila, R. Statistical analysis of the lithic furniture of the ifri ouberrid site in ain elleuh in the moroccan central middle atlas. *Int. J. Adv. Eng. Res. Sci.* **5** (2018).

6. Tarradell, M. *Avance de la primera campaña de excavaciones en Cat Taht El Gar* (Impr. Cremades, 1955).

7. Tarradell, M. Caf taht el gar, cueva neolítica en la región de tetuán (marruecos). *Empúries: revista de món clàssic i antiguitat tardana* 137–166 (1957).

8. Daugas, J. P. & El Idrissi, A. Neolítico antiguo de marruecos en su contexto regional. *Las ocupaciones humanas de la cueva de Caf That el Ghar (Tetuán). Los productos arqueológicos en el contexto del Estrecho de Gibraltar* 63–91 (2008).

9. El Idrissi, A. Le néolithique ancien du maroc septentrional dans son contexte régional. *Rabat: Inst. Natl. des Sci. de l'Archéologie et du Patrimoine* (2001).

10. El Idrissi, A. Le néolithique du maroc: État de la question. *Rubricatum: revista del Museu de Gavà* 333–342 (2012).

11. Daugas, J.-P. Le néolithique du maroc, 25 ans de coopération franco-marocaine. évolution des concepts, bilan documentaire et perspectives de recherche. *Les nouvelles de l'archéologie* 116–121 (2010).

12. Ballouche, A., Ouchaou, B. & El Idrissi, A. More on neolithisation process within the alboran territory. reply to linstädter et al.(in press) in quaternary international. *Quat. Int.* **274**, 175e176 (2012).

13. Ballouche, A. & Marinval, P. Données palynologiques et carpologiques sur la domestication des plantes et l'agriculture dans le néolithique ancien du maroc septentrional. le site de kaf taht el-ghar. *Archeosciences, revue d'Archéométrie* **27**, 49–54 (2003).

14. Morales, J. *et al.* The introduction of south-western asian domesticated plants in north-western africa: an archaeobotanical contribution from neolithic morocco. *Quat. Int.* **412**, 96–109 (2016).

15. Martínez Sánchez, R. M. *et al.* Revisiting the epipalaeolithic-neolithic transition in the extreme nw of africa: the latest results of the chronological sequence of the cave of kaf taht el-ghar (tétouan, morocco). *Afr. Archaeol. Rev.* **38**, 251–274 (2021).

16. Martinez-Sanchez, R. M., Vera-Rodriguez, J. C., Perez-Jorda, G. & Pena-Chocarro, L. Pottery decoration in the neolithic of kaf taht el-ghar (tetouan, morocco). agriwestmed works 2012/la decoracion ceramica en el neolitico de kaf taht el-ghar (tetuan, marruecos). campana agriwestmed 2012. *Zephyrus* 33–62 (2021).

17. Bokbot, Y. & Ben-Nçer, A. Découvertes campaniformes récentes dans les plateaux de zemmour (maroc). In *Proceedings of the 10th Meeting Archéologie et Gobelets*, 12–15 (2008).

18. Ben-Ncer, A., Bokbot, Y., Amani, F. & Ouachi, M. Study of the chalcolithic burial 2 and 3 of ifri n'amr ou moussa (morocco). *Around Petit-Chasseur Site Sion (Valais, Switzerland) New Approaches to Bell Beaker Cult. Archaeopress Archaeol. Oxford, UK* 251–258 (2011).

19. Fregel, R. *et al.* Ancient genomes from north africa evidence prehistoric migrations to the maghreb from both the levant and europe. *Proc. Natl. Acad. Sci.* **115**, 6774–6779 (2018).

20. Turek, J. & Vintr, J. Neolit maghrebu ve světle novỳch radiokarbonovỳch dat. *Živá archeologie* **18**, 10–15 (2016).

21. Laviano, F. La faune neolithique du site d'ifri n'amr o'moussa (oued beth, plateau de zemmour, maroc): methodologie appliquee a une stratigraphie perturbee. *Unpubl. Master's Thesis. Univ. Paul Valery Montpellier: Montpellier, France* (2015).

22. Martínez-Sánchez, R. M., Vera-Rodríguez, J. C., Pérez-Jordà, G., Peña-Chocarro, L. & Bokbot, Y. The beginning of the neolithic in northwestern morocco. *Quat. Int.* **470**, 485–496 (2018).

23. Daugas, J.-P., Texier, P.-J., Raynal, J.-P. & Ballouche, A. Nouvelles données sur le néolithique marocain et ses paléoenvironnements: l'habitat cardial des grottes d'el khril à achakar (province de tanger) et la nécropole néolithique final de rouazi à skhirat (province de skhirat). In *10° Réunion annuelle des Sciences de la Terre*, 167 (Société géologique de France, 1984).

24. Debénath, A., Raynal, J. & Texier, J. Fouilles du cap chatelier in: Activités de la mission préhistorique et paléontologique française au maroc. années 1981–1982. *Bull. d'Archéologie Marocaine* **15**, 18–20 (1984).

25. Texier, J.-P., Raynal, J.-P., Lefèvre, D. & Daugas, J.-P. Le site néolithique de rouazi à skhirat (maroc): contexte stratigraphique et évolution morphodynamique de son environnement littoral. *Quaternaire. Revue de l'Association française pour l'étude du Quaternaire* **19**, 239–247 (2008).

26. Lacombe, J.-P., Daugas, J. P., Murail, P., Cochard, D. & Sbihi-Alaoui, F. Z. La nécropole néolithique de skhirat (maroc): caractérisation de la population inhumée et organisation spatiale. *BMSAP* **26**, 78–87 (2014).

27. Daugas, J.-P. *Le Néolithique du Maroc: pour un modèle d'évolution chronologique et culturelle* (2002).

28. Turek, J. Origin of the bell beaker phenomenon. the moroccan connection. *Backgr. to Beakers. Inq. into regional cultural backgrounds Bell Beaker complex* 191–203 (2012).

29. de Wailly, A. Le kef el baroud et l'ancienneté de l'introduction du cuivre au maroc. *Bull. d'Archéologie Marocaine Rabat* **10**, 47–51 (1976).

30. Ouchaou, B., Amani, F. & Mouhsine, T. Etude archéozoologique du gisement de kehf-el-baroud. *Préhistoire anthropologie méditerranéennes* **7**, 27–38 (1998).

31. Banerjee, A., Dindorf, W., Mikdad, A., Reischmann, T. & Schuhmacher, T. X. Die elfenbeinfunde aus kehfel-baroud (ziaida, ben slimane, marokko) und die frage des nordafrikanischen elefanten. *Madrider Mitteilungen* 113–138 (2011).

32. Martín-Socas, D., Massieu, M. D. C., Herrero, J. L. C. & Rodríguez-Santos, F. J. The beginning of the neolithic in andalusia. *Quat. Int.* **470**, 451–471 (2018).

33. Bernabeu Auban, J. & Pardo-Gordó, S. La impressa en la península ibérica: żespejismo o realidad? una reflexión a partir del binomio radiocarbono-cerámica. *Context. la cerámica impressa: Horizontes culturales en la ınsulaIbérica* 47–57 (2020).

34. Zilhão, J. Early prehistoric navigation in the western mediterranean: Implications for the neolithic transition in iberia and the maghreb. *Eurasian Prehistory. Island Archaeol. Orig. Seafar. East. Mediterr.* **11**, 185–200 (2014).

35. Martins, H. *et al.* Radiocarbon dating the beginning of the neolithic in iberia: new results, new problems. *J. Mediterr. Archaeol.* **28**, 105–131 (2015).

36. Pardo-Gordó, S. ¿ efecto de los huesos de ovicaprinos domésticos en las fechas radiocarbónicas? un primer ensayo metodológico a partir de los datos disponibles en relación con las primeras sociedades neolíticas de la península ibérica. *Arch. de prehistoria levantina* **33** (2020).

37. Morales, J. *et al.* The origins of agriculture in north-west africa: macro-botanical remains from epipalaeolithic and early neolithic levels of ifri oudadane (morocco). *J. Archaeol. Sci.* **40**, 2659–2669 (2013).

38. Linstädter, J. Le site néolithique de l'abri d'hassi ouenzga (rif oriental, maroc). *Beiträge zur allgemeinen und vergleichenden Archäologie* **23**, 85–138 (2003).

39. Martinez Sanchez, R. M. *et al.* The middle neolithic of Morocco's north-western Atlantic strip: New evidence from the el-khil caves (Tangier). *Afr. Archaeol. Rev.* **35**, 417–442 (2018).

40. Caneva, I. Avant l´impresso-cardial: la céramique imprimdu levant et d´afrique du nord au VIIe mllénaire aec. In Binder, D. & Manen, C. (eds.) *Céramiques imprimées de Méditerranée occidentale (VIe millénaire AEC) : données, approches et en jeux nouveaux / Western Mediterranean Impressed Wares (6th millennium BCE): New data, approaches and challenges*, 13–25 (Actes de la séance de la Société préhistorique française de Nice, pp. 13-25, Société Préhistorique Française, 2022).

41. Linstädter, J. Climate induced mobility and the missing middle neolithic of morocco. *Palaeoenvironment development early settlements* 63–80 (2016).

42. Reimer, P. J. *et al.* The intcal20 northern hemisphere radiocarbon age calibration curve (0–55 cal kbp). *Radiocarbon* **62**, 725–757 (2020).

43. Damgaard, P. B. *et al.* Improving access to endogenous dna in ancient bones and teeth. *Sci. reports* **5**, 11184 (2015).

44. Yang, D. Y., Eng, B., Waye, J. S., Dudar, J. C. & Saunders, S. R. Improved dna extraction from ancient bones using silica-based spin columns. *Am. J. Phys. Anthropol. The Off. Publ. Am. Assoc. Phys. Anthropol.* **105**, 539–543 (1998).

45. Günther, T. *et al.* Ancient genomes link early farmers from atapuerca in spain to modern-day basques. *Proc. Natl. Acad. Sci.* **112**, 11917–11922 (2015).

46. Svensson, E. *et al.* Genome of peştera muierii skull shows high diversity and low mutational load in pre-glacial europe. *Curr. Biol.* **31**, 2973–2983 (2021).

47. Meyer, M. & Kircher, M. Illumina sequencing library preparation for highly multiplexed target capture and sequencing. *Cold Spring Harb. Protoc.* **2010**, pdb–prot5448 (2010).

48. Sawyer, S., Krause, J., Guschanski, K., Savolainen, V. & Pääbo, S. Temporal patterns of nucleotide misincorporations and dna fragmentation in ancient dna. *PloS one* **7**, e34131 (2012).

49. Günther, T. *et al.* Population genomics of mesolithic scandinavia: Investigating early postglacial migration routes and high-latitude adaptation. *PLoS biology* **16**, e2003703 (2018).

50. Dabney, J. *et al.* Complete mitochondrial genome sequence of a middle pleistocene cave bear reconstructed from ultrashort dna fragments. *Proc. Natl. Acad. Sci.* **110**, 15758–15763 (2013).

51. Allentoft, M. E. *et al.* Population genomics of bronze age eurasia. *Nature* **522**, 167 (2015).

52. Kircher, M. Analysis of high-throughput ancient dna sequencing data. In *Ancient DNA*, 197–228 (Springer, 2012).

53. Schubert, M., Lindgreen, S. & Orlando, L. Adapterremoval v2: rapid adapter trimming, identification, and read merging. *BMC research notes* **9**, 88 (2016).

54. Li, H. & Durbin, R. Fast and accurate short read alignment with burrows–wheeler transform. *bioinformatics* **25**, 1754–1760 (2009).

55. Skoglund, P. *et al.* Genomic diversity and admixture differs for stone-age scandinavian foragers and farmers. *Science* **344**, 747–750 (2014).

56. Lazaridis, I. *et al.* Ancient human genomes suggest three ancestral populations for present-day europeans. *Nature* **513**, 409 (2014).

57. Green, R. E. *et al.* A complete neandertal mitochondrial genome sequence determined by high-throughput sequencing. *Cell* **134**, 416–426 (2008).

58. Fu, Q. *et al.* A revised timescale for human evolution based on ancient mitochondrial genomes. *Curr. biology* **23**, 553–559 (2013).

59. Rasmussen, M. *et al.* An aboriginal australian genome reveals separate human dispersals into asia. *Science* **334**, 94–98 (2011).

60. Jun, G. *et al.* Detecting and estimating contamination of human dna samples in sequencing and array-based genotype data. *The Am. J. Hum. Genet.* **91**, 839–848 (2012).

61. Schiffels, S. *et al.* Iron age and anglo-saxon genomes from east england reveal british migration history. *Nat. communications* **7**, 10408 (2016).

62. Skoglund, P., Storå, J., Götherström, A. & Jakobsson, M. Accurate sex identification of ancient human remains using dna shotgun sequencing. *J. Archaeol. Sci.* **40**, 4477–4482 (2013).

63. Fu, Q. *et al.* The genetic history of ice age europe. *Nature* **534**, 200 (2016).

64. Li, H. *et al.* The sequence alignment/map format and samtools. *Bioinformatics* **25**, 2078–2079 (2009).

65. Danecek, P. *et al.* The variant call format and vcftools. *Bioinformatics* **27**, 2156–2158 (2011).

66. Weissensteiner, H. *et al.* Haplogrep 2: mitochondrial haplogroup classification in the era of high-throughput sequencing. *Nucleic acids research* **44**, W58–W63 (2016).

67. Van Oven, M. Phylotree build 17: Growing the human mitochondrial dna tree. *Forensic Sci. Int. Genet. Suppl. Ser.* **5**, e392–e394 (2015).

68. Pennarun, E. *et al.* Divorcing the late upper palaeolithic demographic histories of mtdna haplogroups m1 and u6 in africa. *BMC evolutionary biology* **12**, 1–12 (2012).

69. Maca-Meyer, N., González, A. M., Larruga, J. M., Flores, C. & Cabrera, V. M. Major genomic mitochondrial lineages delineate early human expansions. *BMC genetics* **2**, 1–8 (2001).

70. González, A. M. *et al.* Mitochondrial lineage m1 traces an early human backflow to africa. *BMC genomics* **8**, 1–12 (2007).

71. Szécsényi-Nagy, A. *et al.* Tracing the genetic origin of europe's first farmers reveals insights into their social organization. *Proc. Royal Soc. B: Biol. Sci.* **282**, 20150339 (2015).

72. Olalde, I. *et al.* The beaker phenomenon and the genomic transformation of northwest europe. *Nature* **555**, 190 (2018).

73. Marcus, J. H. *et al.* Genetic history from the middle neolithic to present on the mediterranean island of sardinia. *Nat. communications* **11**, 1–14 (2020).

74. Yu, H. *et al.* Genomic and dietary discontinuities during the mesolithic and neolithic in sicily. *Iscience* **25**, 104244 (2022).

75. Harney, É. *et al.* Ancient dna from chalcolithic israel reveals the role of population mixture in cultural transformation. *Nat. communications* **9**, 1–11 (2018).

76. Prendergast, M. E. *et al.* Ancient dna reveals a multistep spread of the first herders into sub-saharan africa. *Science* **365**, eaaw6275 (2019).

77. Valdiosera, C. *et al.* Four millennia of iberian biomolecular prehistory illustrate the impact of prehistoric migrations at the far end of eurasia. *Proc. Natl. Acad. Sci.* **115**, 3428–3433 (2018).

78. Mathieson, I. *et al.* The genomic history of southeastern europe. *Nature* **555**, 197 (2018).

79. Lipson, M. *et al.* Parallel palaeogenomic transects reveal complex genetic history of early european farmers. *Nature* **551**, 368 (2017).

80. Lazaridis, I. *et al.* Genomic insights into the origin of farming in the ancient near east. *Nature* **536**, 419 (2016).

81. Fu, Q. *et al.* Genome sequence of a 45,000-year-old modern human from western siberia. *Nature* **514**, 445–449 (2014).

82. Raghavan, M. *et al.* Upper palaeolithic siberian genome reveals dual ancestry of native americans. *Nature* **505**, 87–91 (2014).

83. Jones, E. R. *et al.* Upper palaeolithic genomes reveal deep roots of modern eurasians. *Nat. communications* **6**, 1–8 (2015).

84. Mathieson, I. *et al.* Genome-wide patterns of selection in 230 ancient eurasians. *Nature* **528**, 499 (2015).

85. Olalde, I. *et al.* Derived immune and ancestral pigmentation alleles in a 7,000-year-old mesolithic european. *Nature* **507**, 225 (2014).

86. Olalde, I. *et al.* The genomic history of the iberian peninsula over the past 8000 years. *Science* **363**, 1230–1234 (2019).

87. González-Fortes, G. *et al.* Paleogenomic evidence for multi-generational mixing between neolithic farmers and mesolithic hunter-gatherers in the lower danube basin. *Curr. Biol.* **27**, 1801–1810 (2017).

88. Villalba-Mouco, V. *et al.* Survival of late pleistocene hunter-gatherer ancestry in the iberian peninsula. *Curr. Biol.* (2019).

89. Feldman, M. *et al.* Late pleistocene human genome suggests a local origin for the first farmers of central anatolia. *Nat. Commun.* **10**, 1218 (2019).

90. Kılınç, G. M. *et al.* The demographic development of the first farmers in anatolia. *Curr. Biol.* **26**, 2659–2666 (2016).

91. Hofmanová, Z. *et al.* Early farmers from across europe directly descended from neolithic aegeans. *Proc. Natl. Acad. Sci.* **113**, 6886–6891 (2016).

92. Omrak, A. *et al.* Genomic evidence establishes anatolia as the source of the european neolithic gene pool. *Curr. Biol.* **26**, 270–275 (2016).

93. Lazaridis, I. *et al.* Genetic origins of the minoans and mycenaeans. *Nature* **548**, 214 (2017).

94. Olalde, I. *et al.* A common genetic origin for early farmers from mediterranean cardial and central european lbk cultures. *Mol. biology evolution* **32**, 3132–3142 (2015).

95. Martiniano, R. *et al.* The population genomics of archaeological transition in west iberia: Investigation of ancient substructure using imputation and haplotype-based methods. *PLoS genetics* **13**, e1006852 (2017).

96. Rivollat, M. *et al.* Ancient genome-wide dna from france highlights the complexity of interactions between mesolithic hunter-gatherers and neolithic farmers. *Sci. advances* **6**, eaaz5344 (2020).

97. Schuenemann, V. J. *et al.* Ancient egyptian mummy genomes suggest an increase of sub-saharan african ancestry in post-roman periods. *Nat. communications* **8**, 15694 (2017).

98. Rodríguez-Varela, R. *et al.* Genomic analyses of pre-european conquest human remains from the canary islands reveal close affinity to modern north africans. *Curr. Biol.* **27**, 3396–3402 (2017).

99. Schlebusch, C. M. *et al.* Southern african ancient genomes estimate modern human divergence to 350,000 to 260,000 years ago. *Science* **358**, 652–655 (2017).

100. Skoglund, P. *et al.* Reconstructing prehistoric african population structure. *Cell* **171**, 59–71 (2017).

101. Llorente, M. G. *et al.* Ancient ethiopian genome reveals extensive eurasian admixture in eastern africa. *Science* **350**, 820–822 (2015).

102. Antonio, M. L. *et al.* Ancient rome: A genetic crossroads of europe and the mediterranean. *Science* **366**, 708–714 (2019).

103. Lipson, M. *et al.* Ancient west african foragers in the context of african population history. *Nature* **577**, 665–670 (2020).

104. Mallick, S. *et al.* The simons genome diversity project: 300 genomes from 142 diverse populations. *Nature* **538**, 201–206 (2016).

105. Monroy Kuhn, J. M., Jakobsson, M. & Günther, T. Estimating genetic kin relationships in prehistoric populations. *PloS one* **13**, e0195491 (2018).

106. Patterson, N., Price, A. L. & Reich, D. Population structure and eigenanalysis. *PLoS genetics* **2**, e190 (2006).

107. R Development Core Team. *R: A Language and Environment for Statistical Computing*. R Foundation for Statistical Computing, Vienna, Austria (2008). URL http://www.R-project.org. ISBN 3-900051-07-0.

108. Wickham, H. *ggplot2: Elegant Graphics for Data Analysis* (Springer-Verlag New York, 2016). URL https://ggplot2.tidyverse.org.

109. Alexander, D. H., Novembre, J. & Lange, K. Fast model-based estimation of ancestry in unrelated individuals. *Genome research* **19**, 1655–1664 (2009).

110. Purcell, S. *et al.* Plink: a tool set for whole-genome association and population-based linkage analyses. *The Am. journal human genetics* **81**, 559–575 (2007).

111. Behr, A. A., Liu, K. Z., Liu-Fang, G., Nakka, P. & Ramachandran, S. pong: Fast analysis and visualization of latent clusters in population genetic data. *Bioinformatics* **32**, 2817–2823 (2016).

112. Skoglund, P. *et al.* Genetic evidence for two founding populations of the americas. *Nature* **525**, 104 (2015).

113. Patterson, N. *et al.* Ancient admixture in human history. *Genetics* **192**, 1065–1093 (2012).

114. Haak, W. *et al.* Massive migration from the steppe was a source for indo-european languages in europe. *Nature* **522**, 207 (2015).

115. Villalba-Mouco, V. *et al.* Genomic transformation and social organization during the copper age–bronze age transition in southern iberia. *Sci. advances* **7**, eabi7038 (2021).

116. Harney, E., Patterson, N., Reich, D. & Wakeley, J. Assessing the performance of qpadm: a statistical tool for studying population admixture. *Genetics* **217**, iyaa045 (2021).

117. Maier, R., Flegontov, P., Flegontova, O., Changmai, P. & Reich, D. On the limits of fitting complex models of population history to genetic data. *bioRxiv* (2022).

118. Loh, P.-R. *et al.* Inferring admixture histories of human populations using linkage disequilibrium. *Genetics* **193**, 1233–1254 (2013).

119. Narasimhan, V. M. *et al.* The formation of human populations in south and central asia. *Science* **365**, eaat7487 (2019).

120. Gamba, C. *et al.* Genome flux and stasis in a five millennium transect of european prehistory. *Nat. communications* **5**, 1–9 (2014).

121. Consortium, . G. P. *et al.* A global reference for human genetic variation. *Nature* **526**, 68 (2015).

122. Ceballos, F. C., Joshi, P. K., Clark, D. W., Ramsay, M. & Wilson, J. F. Runs of homozygosity: windows into population history and trait architecture. *Nat. Rev. Genet.* **19**, 220–234 (2018).

123. Chang, C. C. *et al.* Second-generation plink: rising to the challenge of larger and richer datasets. *Gigascience* **4**, s13742–015 (2015).

124. Schiffels, S. & Durbin, R. Inferring human population size and separation history from multiple genome sequences. *Nat. genetics* **46**, 919–925 (2014).

125. Chaitanya, L. *et al.* The hirisplex-s system for eye, hair and skin colour prediction from dna: Introduction and forensic developmental validation. *Forensic Sci. Int. Genet.* **35**, 123–135 (2018).

126. Walsh, S. *et al.* The hirisplex system for simultaneous prediction of hair and eye colour from dna. *Forensic Sci. Int. Genet.* **7**, 98–115 (2013).

127. Crawford, N. G. *et al.* Loci associated with skin pigmentation identified in african populations. *Science* **358**, eaan8433 (2017).

128. Martin, A. R. *et al.* An unexpectedly complex architecture for skin pigmentation in africans. *Cell* **171**, 1340–1353 (2017).
