# Supplementary material for: Northwest African Neolithic initiated by migrants from Iberia and Levant
Source: Nature. 2023 Jun 7;618(7965):550–6. doi: 10.1038/s41586-023-06166-6 (PMC10266975; doi:10.1038/s41586-023-06166-6)
Supplement: Supplementary file 2 — Reporting Summary [file 41586_2023_6166_MOESM2_ESM.pdf]

Corresponding author(s): Mattias Jakobsson

Last updated by author(s): 14/04/23

Reporting Summary

Nature Portfolio wishes to improve the reproducibility of the work that we publish. This form provides structure for consistency and transparency in reporting. For further information on Nature Portfolio policies, see our Editorial Policies and the Editorial Policy Checklist.

Statistics

For all statistical analyses, confirm that the following items are present in the figure legend, table legend, main text, or Methods section.

n/a	Confirmed	
☐	☒	The exact sample size (*n*) for each experimental group/condition, given as a discrete number and unit of measurement
☐	☒	A statement on whether measurements were taken from distinct samples or whether the same sample was measured repeatedly
☐	☒	The statistical test(s) used AND whether they are one- or two-sided<br>*Only common tests should be described solely by name; describe more complex techniques in the Methods section.*
☒	☐	A description of all covariates tested
☒	☐	A description of any assumptions or corrections, such as tests of normality and adjustment for multiple comparisons
☐	☒	A full description of the statistical parameters including central tendency (e.g. means) or other basic estimates (e.g. regression coefficient) AND variation (e.g. standard deviation) or associated estimates of uncertainty (e.g. confidence intervals)
☐	☒	For null hypothesis testing, the test statistic (e.g. *F*, *t*, *r*) with confidence intervals, effect sizes, degrees of freedom and *P* value noted<br>*Give P values as exact values whenever suitable.*
☒	☐	For Bayesian analysis, information on the choice of priors and Markov chain Monte Carlo settings
☒	☐	For hierarchical and complex designs, identification of the appropriate level for tests and full reporting of outcomes
☒	☐	Estimates of effect sizes (e.g. Cohen's *d*, Pearson's *r*), indicating how they were calculated

Our web collection on statistics for biologists contains articles on many of the points above.

Software and code

Policy information about availability of computer code

Data collection	Sequence demultiplexing: MergeReadsFastQ_cc.py, Adapter Removal v2.1.7.<br>Simons Genome Diversity Project datasets (https://www.simonsfoundation.org/simons-genome-diversity-project/) available on UPPMAX.<br>Comparative ancient individuals' genomic data downloaded from the European Nucleotide Archive (ENA), under the accession numbers provided in the references listed in Supplementary Data File 2.
Data analysis	A full description of all software and respective packages used for data analysis can be found in the Supplementary Information document and are publicly available. For genomic reads mapping: Burrows-Wheller Aligner (BWA, v. 0.7.13); genomic libraries merging: samtools v. 1.5. mtDNA contamination estimates: contamMix (1.0-10); X-chromosome contamination estimates: ANGSD v. 0.902; autosomal contamination estimates: verifyBamID v.1.1.2. Mt haplogroup assignement: Haplogrep v. 2.1.16 and PhyloTree mtDNA tree Build 17 (18 Feb 2016); Y chromosome haplogroup assignement: ISOGG (10, April 21, 2016) SNPs called using samtools v. 1.5. Pseudohaploid genomic dataset management (including LD pruning and datasets merging): PLINK v. 1.9. Kinship analysis: READ. PCA: smartpca v.10210 (EIGENSOFT package); model-based clustering analysis: ADMIXTURE v. 1.3.0 and PONG v. 1.5. f -statistics: python script POPSTATS (https://github.com/pontussk/popstats); Admixture modelling: qpAdm (ADMIXTOOLS v. 5.0) via qpAdm_wrapper (https://github.com/pontussk/qpAdm_wrapper); Admixture graphs: ADMIXTOOLS2 findGraphs function. Admixture dating: ALDER v. 1.03 and DATES v. 753. Diploid genotype calling GATK v. 3.5.0. Diploid genomic dataset management (including SNP selection) Vcftools v. 0.1.16 and Plink v. 1.9. Runs of Homozygosity: Plink v. 1.9. Pairwise Sequentially Markovian Coalescent (PSMC) implemented on MSMC v. 0.1.0. Phenotypic variation analysis: ANGSD v. 0.933. Results visualization and plot generation: R v. 3.4.067, ggplot2. Radiocarbon dates calibration: Oxcal v4.4 and IntCal20.

For manuscripts utilizing custom algorithms or software that are central to the research but not yet described in published literature, software must be made available to editors and reviewers. We strongly encourage code deposition in a community repository (e.g. GitHub). See the Nature Portfolio guidelines for submitting code & software for further information.

Data

Policy information about availability of data

All manuscripts must include a data availability statement. This statement should provide the following information, where applicable:

- Accession codes, unique identifiers, or web links for publicly available datasets
- A description of any restrictions on data availability
- For clinical datasets or third party data, please ensure that the statement adheres to our policy

Human reference genome build 37 (hs37d5) (https://ftp.1000genomes.ebi.ac.uk/vol1/ftp/technical/reference/phase2_reference_assembly_sequence/)
All the generated sequence data are available as bamfiles of aligned reads at the European Nucleotide Archive (ENA) under the accession number PRJEB59008.

Human research participants

Policy information about studies involving human research participants and Sex and Gender in Research.

Reporting on sex and gender	N/A
Population characteristics	N/A
Recruitment	N/A
Ethics oversight	N/A

Note that full information on the approval of the study protocol must also be provided in the manuscript.

Field-specific reporting

Please select the one below that is the best fit for your research. If you are not sure, read the appropriate sections before making your selection.

☒ Life sciences ☐ Behavioural & social sciences ☐ Ecological, evolutionary & environmental sciences

For a reference copy of the document with all sections, see nature.com/documents/nr-reporting-summary-flat.pdf

Life sciences study design

All studies must disclose on these points even when the disclosure is negative.

Sample size	Genomic and radiocarbon data from nine ancient individuals from Morocco were analysed in this study. The sample size was dependent on the availability of human remains dating to the Stone Age from northwestern Africa, with preserved and retrievable ancient DNA sequences. These specimens are very rare, given the poor molecular preservation of human remains from this period in that region. Given the millions of genetic variants analysed for each individual, information about the genetic history can be retrieved.
Data exclusions	Reads shorter than 35 base pairs (bp), with more than 10% mismatch from the Reference genome and mapping quality score below 30 were discarded while preparing bamfiles for merged genomic libraries data. For samples not subjected to Uracil-Specific Excision Reagent (USER) treatment, 10 bp at the reads ends were excluded. For samples with partial treatment (comparative dataset) 2 bp were trimmed off of the reads ends. For analyses, minimum mapping and read qualities were set to 30. Pseudohaploid dataset was generated by randomly drawing one read at each SNP site, and that allele assumed to be homozygous. LD pruning for ADMIXTURE resulted in a reduction of the number of analysed SNPs (originally 1,379,466) to 812,092. When pairs of first-degree relatives (of comparative populations) were found, the individual with lower genomic coverage of the pair was excluded from analysis. Diploid dataset was generated with samples with a minimum of 9x genomic coverage. For MSMC's implementation of PSMC', minimum mapping quality of 30 and minimum genotype quality of 50 were used. For phenotypic analysis, genotype likelihoods were computed based on minimum mapping and read quality of 30 and read depth of 5.
Replication	Several DNA extracts and multiple genomic libraries were generated for each sample (as reported in Table S1), and several rounds of sequencing were performed for each library (as reported in Supplementary Data File 15) as replication. Data was merged for downstream analysis after confirming similar results, as expected of different replicates of the same individual's genomic data, such as contamination estimates, mitochondrial haplogroup. Thousands to millions of genetic markers were then analysed as an internal replication of the results. Detailed description of the methods used, including samples included in the dataset, software employed and respective parameters is available in the Supplementary Information.
Randomization	Randomization is not applicable to this study. Samples were grouped according to the archaeological site of origin and radiocarbon date. Groups are validated by verifying genetic affinities among its several individuals.
Blinding	Blinding is not applicable to this study. The archaeological context, including site location and estimated date, of each individual analysed is known prior to sampling and analysis, as these are relevant for conceiving the study.

Reporting for specific materials, systems and methods

We require information from authors about some types of materials, experimental systems and methods used in many studies. Here, indicate whether each material, system or method listed is relevant to your study. If you are not sure if a list item applies to your research, read the appropriate section before selecting a response.

Materials & experimental systems

n/a	Involved in the study
☒	☐ Antibodies
☒	☐ Eukaryotic cell lines
☐	☒ Palaeontology and archaeology
☐	☒ Animals and other organisms
☒	☐ Clinical data
☒	☐ Dual use research of concern

Methods

n/a	Involved in the study
☒	☐ ChIP-seq
☒	☐ Flow cytometry
☒	☐ MRI-based neuroimaging

Palaeontology and Archaeology

Specimen provenance	Archaeological samples were excavated in Morocco at the archaeological sites of Ifri Ouberrid, Kaf Taht el Ghar, Ifri n'Amr ou Moussa and Skhirat-Rouazi Necropolis. Appropriate permits were obtained to conduct sampling and export archaeological material from the Institut National des Sciences de l´Archèologie et du Patrimoine (INSAP) in Rabat, Morocco.
Specimen deposition	The Institut National des Sciences de l´Archèologie et du Patrimoine (INSAP) in Rabat, Morocco is the sole curator of the specimens.
Dating methods	All individuals were directly radiocarbon dated using accelerator mass spectrometry (AMS) at the Tandem Laboratory at Ångström, Uppsala, except for ktg001, which was dated at the Beta Analytic Carbon dating laboratory and iam004, which date was obtained from the literature. Radiocarbon calibration was performed using OxCal v.4.4 and the IntCal20 dataset.

☒ Tick this box to confirm that the raw and calibrated dates are available in the paper or in Supplementary Information.

Ethics oversight	Permits for sampling and analyses of the archaeological material were obtained from the appropriate institutions.

Note that full information on the approval of the study protocol must also be provided in the manuscript.

Animals and other research organisms

Policy information about studies involving animals; ARRIVE guidelines recommended for reporting animal research, and Sex and Gender in Research

Laboratory animals	n/a
Wild animals	n/a
Reporting on sex	The sex of the individuals for which archaeological remains were analysed was determined based on the ratio of coverage of the X chromosome and Y chromosome relative to the autosomes.
Field-collected samples	n/a
Ethics oversight	n/a

Note that full information on the approval of the study protocol must also be provided in the manuscript.
